# Anthropometric and Metabolic Traits Across Ancestries in the UK Biobank

**DOI:** 10.1155/jobe/5149353

**Published:** 2026-07-21

**Authors:** Delnaz Roshandel, Andrew D. Paterson, Satya Dash

**Affiliations:** ^1^ Department of Genetics, Genetics and Genome Biology Program, The Hospital for Sick Children, Toronto, Ontario, Canada, sickkids.ca; ^2^ Department of Medicine, Divisions of Epidemiology and Biostatistics, Dalla Lana School of Public Health, University of Toronto, Toronto, Ontario, Canada, utoronto.ca; ^3^ Institute of Medical Sciences, Department of Medicine, University of Toronto, Toronto, Ontario, Canada, utoronto.ca; ^4^ Department of Medicine, University Health Network, University of Toronto, Toronto, Ontario, Canada, utoronto.ca

## Abstract

**Aims:**

Increased waist–hip ratio (WHR) increases free fatty acid (FFA) flux, contributing to dysglycemia and dyslipidemia in Europeans. We examined these phenomena in non‐Europeans, a group with higher prevalence of metabolic disease.

**Methods:**

We undertook sex‐stratified analyses of WHR, body composition, HbA1c, lipids and total FFA in people without diabetes (Males: 153,613, Females: 193,574) of European (E, *N* = 333,465), African (A, *N* = 2040), African American (AA, *N* = 2808), South Asian (SA, *N* = 5121), East Asian (EA, *N* = 1374), Asian‐Pacific Islander (API, *N* = 570) and Latin American 1 (LA‐1, Puerto Rican and Dominican, *N* = 1400) and Latin American 2 (LA‐2, Mexican and Mexican American, *N* = 409) ancestry as defined by genetic data in the UK Biobank. Sex‐matched Es were the comparator group for all analyses.

**Results:**

After adjustment for WHR, socioeconomic status, smoking, alcohol use, physical activity and menopause status in females, all groups except LA‐2 males had significantly higher HbA1c (*p* < 2E − 3). Increased FFA and dyslipidemia was only seen in EA and API males but not females. SA males and females had dyslipidemia with no increase in FFA. A and AA males and females had lower FFA and improved lipids, while no significant differences were seen in lipid parameters amongst LA‐1 males and females and LA‐2 females compared to Es.

**Conclusions:**

Dysglycemia is seen in most non‐E ancestries after adjustment for WHR and does not always associate with increased FFA and dyslipidemia. This indicates that factors beyond body composition and lipid flux may contribute to metabolic disease in non‐Es.

## 1. Introduction

The increased prevalence of obesity is a major driver of insulin resistance (IR), dysglycemia and dyslipidemia [1]. Compared to overall adiposity, fat distribution likely better predicts insulin sensitivity and metabolic traits. Increased central fat accumulation and/or reduced leg fat (either measured directly or using the surrogate measure of waist–hip ratio [WHR]) associate with increased IR, dysglycemia and atherogenic dyslipidemia characterized by increased serum triglyceride (TG) and Apolipoprotein B (apoB)‐containing TG rich lipoproteins and reduced high‐density lipoprotein (HDL) [1–4]. Mechanistically, this fat distribution is postulated to represent reduced adipose storage capacity which in the setting of weight gain predisposes to increased visceral adiposity, increased free fatty acid (FFA) flux and ectopic lipid deposition in liver and muscle which impairs insulin signalling [1–4]. An extreme example of this phenotype is familial partial lipodystrophy characterized by the absence of leg fat with associated IR, ectopic lipid, dysglycemia and dyslipidemia [5]. In the setting of beta‐cell dysfunction, impaired insulin signalling due to ectopic lipid deposition associates with dysglycemia and Type 2 diabetes. IR‐associated hyperinsulinemia and increased lipid flux due to reduced storage capacity also contribute to increased TG and TG‐rich lipoprotein production from the liver and gut and reduced HDL [6, 7]. Genetic studies, undertaken predominantly in cohorts of European (E) ancestry, implicate central adiposity and reduced leg adiposity as causal risk factors for metabolic disease [2–4].

The prevalence of Type 2 diabetes and metabolic disease is higher in non‐Es and frequently at lower body mass index thresholds [8, 9]. Increased IR may contribute to the higher prevalence of Type 2 diabetes in E ancestry groups, with highest surrogate measures of IR seen in South Asians (SAs), but this is likely not fully explained by differences in fat distribution [10]. Furthermore, although African Americans (AAs) have higher surrogate measures of IR compared to Whites, they paradoxically do not manifest dyslipidemia and have lower TG and apoB and higher HDL [11, 12]. Collectively, this suggests that factors beyond fat distribution and lipid flux may contribute to metabolic disease in non‐E ancestries. Limitations of some of the reports to date include the relatively small sample size in some studies, with limited data on leg fat distribution, apoB and FFA and noncontemporaneous recruitment across groups [10, 11, 13, 14].

Here, we report on differences in anthropometric and body composition measures across ancestry groups (defined by genetic principal components) in the UK Biobank using E ancestry as a comparator. We further assess their association with glycaemic and lipid parameters including FFA and apoB. The rationale for undertaking these observational analyses across ancestral groups is that this will inform subsequent genetic and mechanistic studies. As sex influences fat distribution and metabolic traits, we undertook sex‐stratified analyses. A notable advantage of using the UK Biobank data is the large sample size and contemporaneous collection of data using standardized methods.

## 2. Research Design and Methods

Ethics approval for this study was obtained in The Hospital for Sick Children (HSC #1000073707). This research has been conducted using the UK Biobank Resource under Application Number 48873.

### 2.1. Study Population

All individuals without diabetes from the UK Biobank with genotyping data, which was required for ancestry assignment, were included in this analysis. To identify individuals with diabetes, we used “First Occurrences” from “Health Related Outcomes” containing any code mapped to 3‐character ICD‐10 in primary care data, hospital inpatient data, death register records and self‐reported medical conditions. Any individual with insulin‐dependent diabetes mellitus (Unique Data Identifier [UDI]: 130706 and 130707), non‐insulin‐dependent diabetes mellitus (UDI: 130708 and 130709), malnutrition‐related diabetes mellitus (UDI: 130710 and 130711), other specified diabetes mellitus (UDI: 130712 and 130713) and unspecified diabetes mellitus (UDI: 130714 and 130715) were excluded from the analysis. Any individual with glucose (UDI: p30740_i0) ≥ 7 mmol/L and HbA1c (UDI: p30750_i0) ≥ 48 mmol/L were also excluded (Table S1). Overall diabetes prevalence was highest in SA (32%), followed by African (A) (24%), AA (22%), Asian‐Pacific Islander (API) (17%), Latin‐American 1(16%), East Asian (EA) (13%), E (11%) and Latin‐American 2 (10%). Genetic sex (UDI 22001‐0.0) was used to assign individuals to male and female groups. Menopausal status in females was defined using self‐reported data (UDI: p2724_i0) and operative procedure records coded according to the Office of Population Censuses and Surveys Classification of Interventions and Procedures (OPCS‐3, UDI: 41273, and OPCS‐4, UDI: 41272). Participants were classified as having reached menopause if they self‐reported menopause (“Yes”) or had evidence of bilateral oophorectomy based on OPCS‐3 or OPCS‐4 procedure codes prior to Assessment 0 (baseline).

### 2.2. Ancestry Assignment

The centrally imputed genotype data from the UK Biobank Axiom arrays was used. GrafPop v1.0 [15, 16], which leverages ∼100k ancestry related single nucleotide polymorphisms (SNPs) was used to infer ancestries: E, A, AA, API, EA, Latin American 1 (LA‐1; Puerto Rican/Dominican), Latin American 2 (LA‐2, Mexican/Latino) and SA. Individuals with mixed ancestry (Other) were excluded.

### 2.3. Validating Bioimpedance Measurements

Body fat percentage (BFP; UDI: p23281_i2), trunk fat percentage (TFP; UDI: p23286_i2) and leg fat percentage (LFP; UDI: p23276_i2) were measured by DXA in a subset of individuals at Assessment 2 (2014+, https://biobank.ndph.ox.ac.uk/showcase/refer.cgi?id%3D502). These participants also have bioimpedance data (UDI: p23099_i2, p23127_i2, p23115_i2 and p23111_i2) at this assessment (*N* = 45,203; https://biobank.ndph.ox.ac.uk/showcase/refer.cgi?id%3D1421). Bioimpedance LFP was calculated by averaging left and right LFPs. We checked concordance between bioimpedance and DXA measurements to validate bioimpedance measured BFP, TFP and LFP.

### 2.4. Anthropometric and Body Composition Measures

Measurements at baseline (Assessment 0) were used. WHR was calculated by dividing waist circumference (UDI: p48_i0) by hip circumference (UDI: p49_i0). Individuals with WHRs < 0.25 or > 2 were excluded. BFP (UDI: p23099_i0), TFP (UDI: p23127_i0) and left and right LFPs (UDI: p23115_i0 and p23111_i0) were extracted from bioimpedance data. LFP was calculated by averaging left and right LFP. Individuals with > 20% difference in their right and left LFPs were excluded. Plasma leptin levels were extracted from OLINK proteomics data obtained from 53,039 participants (data coding: 143) [17].

### 2.5. Glycaemic and Lipid Traits

Nonfasting serum glucose (UDI: p30740_i0), HbA1c (UDI: p30750_i0), TG (UID: p30870_i0), HDL (UID: p30760_i0), apoB (UID: p30640_i0) and FFA (UID: p23442_i0, plasma nuclear magnetic resonance (NMR) metabolomics [18] available in almost half of the individuals) measured at baseline were used. Individuals on lipid lowering medications (gemfibrozil, gemfibrozil product, fenofibrate, clofibrate, bezafibrate, cholestyramine, cholestyramine product, cholestyramine + aspartame, colesevelam, bezalip, bezalip‐mono, questran, niacin, simvastatin, atorvastatin, rosuvastatin, fluvastatin and lipostat) were excluded from TG, HDL, apoB and FFA analyses (Table S1).

### 2.6. Statistical Analysis

Baseline characteristics were compared between each ancestry and Es, stratified by sex, using the *t*‐test for age at recruitment (UID: p21022); the Wilcoxon rank‐sum test for Townsend depravation index (UID: p22189_i0) and summed metabolic equivalent task (MET) minutes per week for all activity (UID: p22040_i0); the Cochran–Armitage trend test for smoking status (UID: p20116_i0) and alcohol intake frequency (UID: p1558_i0); and the chi‐squared test for menopausal status in females.

To validate bioimpedance measurements, intraclass correlation coefficient (ICC) between fat percentages measured by bioimpedance and DXA at Assessment 2 were calculated using ICC implemented in “psych” library in R. ICC3, where a fixed set of k judges rate each target was reported. Age and sex differences in individuals with and without DXA were tested using the two‐sample *t*‐test and chi‐squared test, respectively. Hexbin bivariate density plots were used to visualize the correlation between bio‐impedance and DXA, generated using the lattice and hexbin packages in R.

Association of ancestry with anthropometric and body composition measures (WHR, BFP, TFP, LFP and leptin) was tested using linear regression including age at recruitment, smoking status, alcohol intake frequency, Townsend depravation index and summed MET minutes per week for all activity (UID: p22040_i0) as covariates in the models separately by sex. In females, the menopause status was also included as a covariate in the model. As age is a major predictor of outcomes, we undertook additional analyses of the association of ancestry with anthropometric and metabolic parameters using adding centred quadratic age as a covariate in the model that also includes linear age.

Association of ancestry with glycaemic and lipid traits (glucose, HbA1c, TG, HDL, apoB and FFA) was tested using linear regression with the same covariates as anthropometric and body composition measures plus WHR, fasting time (UDI: 74, Figure S1) dichotomized by median (> 3 h and ≤ 3 h) and the hour when blood sample was collected (UDI: 3166) as covariates in the model.

Since fourteen tests were performed per trait (seven ancestries and two sexes; 7 × 2 = 14), multiple testing–corrected *p* value threshold was set at 3.57 × 10^−3^ (0.05/14).

To compare the effect size of ancestry vs. sex on anthropometric/body composition measures and glycaemic/lipid traits, we repeated the analysis in all including both sexes with the same covariates mentioned above (except for menopause status) plus sex as a covariate in the model. All analyses were performed in R v4.1.3 [19].

## 3. Results

Baseline demographic features for males and females are presented in Tables 1 and 2, respectively (Figures S4 and S5). Genetic distance (GD) scores by ancestries are illustrated in Figures S and S5. E ancestry followed by SA, AA and A had the largest sample sizes in both sexes. Amongst males, EA(*n* = 433), API (*n* = 214) and LA‐2 (*n* = 148), while amongst females, LA‐2 (*n* = 261) had a sample size of < 500. Participants from all other ancestries were generally younger and had higher Townsend deprivation index and lower alcohol intake compared to Es (Tables S2 and S3). Males from all ancestries had lower physical activity (lower MET) compared to Es except for AA and LA‐2, where the difference was not significant. Smoking was less frequent in A, SA and EA males compared to Es, whereas it was more frequent in LA males (Table S2). Frequency of postmenopausal females was significantly lower in all ancestries compared to Es. EA and SA females were less physically active (lower MET) compared to Es, whereas LA‐2 females were more physically active, with no significant difference between other ancestries and Es. Smoking was less frequent in females from all ancestries compared to Es except for both LAs, where it was similar to Es (Table S3).

**TABLE 1:** Male characteristics at baseline.

Ancestry	E	A	AA	EA	API	SA	LA‐1	LA‐2	All
*N*	147,530	912	1073	433	214	2608	695	148	153,613
Age (years)	56.5 (8.2)	49.5 (7.3)	51 (7.9)	51.8 (8.2)	49.8 (8)	52.1 (8.6)	51 (8.1)	51.4 (8.2)	56.3 (8.3)
BFP	24.7 (5.6)	24.8 (5.5)	24.4 (5.6)	20.5 (5.1)	23.6 (4.8)	25.4 (5.0)	24.8 (5.5)	24.8 (5.7)	24.7 (5.6)
TFP	27.0 (6.4)	27.0 (6.4)	26.5 (6.6)	22.7 (6.5)	26.1 (5.9)	28.0 (5.6)	27.1 (6.4)	27.4 (6.4)	27.0 (6.4)
LFP	21.6 (5.0)	21.6 (4.9)	21.6 (5.0)	16.8 (3.9)	19.7 (4.3)	21.8 (4.7)	21.4 (4.9)	21.1 (5.2)	21.5 (5.0)
WHR	0.93 (0.06)	0.90 (0.06)	0.90 (0.06)	0.90 (0.06)	0.92 (0.06)	0.94 (0.06)	0.92 (0.06)	0.94 (0.06)	0.93 (0.06)
Leptin (NPX)									
N	15,228	168	155	40	17	262	97	10	15,977
Mean (SD)	−0.95 (1.11)	−0.99 (1.19)	−1.0 (1.27)	−1.8 (1.09)	−1.3 (0.89)	−0.61 (1.03)	−1.10 (1.09)	−0.83 (1.06)	−0.95 (1.11)
Glucose (mmol/L)	4.92 (0.57)	4.80 (0.58)	4.83 (0.56)	4.99 (0.61)	4.98 (0.59)	4.85 (0.56)	4.85 (0.59)	4.90 (0.52)	4.92 (0.57)
HbA1c (mmol/L)	34.6 (3.6)	36.1 (5.3)	36.6 (4.6)	36.3 (3.9)	36.1 (3.4)	36.4 (4.0)	35.6 (4.1)	35.2 (3.5)	34.7 (3.6)
Triglycerides (mmol/L)^∗^									
*N*	124,783	848	1000	404	195	2159	606	132	130,127
Mean (SD)	1.93 (1.11)	1.28 (0.80)	1.47 (0.94)	2.05 (1.35)	2.19 (1.35)	2.12 (1.20)	1.94 (1.15)	2.16 (1.41)	1.93 (1.12)
HDL (mmol/L)^∗^									
*N*	124,783	848	1000	404	195	2159	606	132	130,127
Mean (SD)	1.31 (0.31)	1.32 (0.32)	1.33 (0.34)	1.28 (0.30)	1.23 (0.27)	1.18 (0.27)	1.19 (0.28)	1.24 (0.34)	1.31 (0.31)
Apolipoprotein B (g/L)^∗^									
*N*	123,993	841	991	399	194	2139	602	130	129,289
Mean (SD)	1.08 (0.23)	1.00 (0.23)	1.02 (0.24)	1.03 (0.23)	1.07 (0.21)	1.09 (0.21)	1.07 (0.23)	1.07 (0.24)	1.07 (0.23)
Free fatty acids (mmol/L)^∗^									
*N*	70,296	420	512	216	100	1164	345	56	73,109
Mean (SD)	12.11 (2.43)	10.21 (2.15)	10.79 (2.14)	11.89 (2.41)	12.55 (2.24)	12.03 (2.41)	11.74 (2.39)	12.25 (3.00)	12.09 (2.44)
Townsend	−1.53 (2.98)	3.13 (3.51)	1.95 (3.52)	−0.04 (3.57)	0.80 (3.32)	0.22 (3.15)	1.34 (3.80)	2.15 (3.95)	−1.43 (3.05)
MET	2842 (2868)	2643 (2918)	2937 (2994)	2248 (2324)	2145 (2147)	2318 (2533)	2677 (3071)	2809 (2777)	2830 (2864)
Smoking status									
Never	73,842 (50%)	630 (69%)	535 (50%)	284 66%)	127 (59%)	1743 (67%)	292 (42%)	52 (35%)	77,505 (50%)
Previous	55,560 (38%)	152 (17%)	276 (26%)	91 21%)	48 (22%)	446 (17%)	225 (32%)	56 (38%	56,854 (37%)
Current	17,582 (11%)	112 (12%)	254 (24%)	53 (12%)	35 (16%)	346 (13%)	166 (24%)	32 (22%)	18,580 (12%)
Alcohol intake									
Never	6685 (5%)	221 (24%)	176 (16%)	80 (18%)	40 (19%)	912 (35%)	217 (31%)	6 (4%)	8337 (5%)
Special occasions	8890 (6%)	228 (25%)	163 (15%)	133 (31%)	40 (19%)	365 (14%)	108 (16%)	29 (20%)	9956 (6%)
1–3 Times per month	12,481 (8%)	98 (11%)	120 (11%)	50 (12%)	17 (8%)	214 (8%)	75 (11%)	23 (16%)	13,078 (9%)
1‐2 Times per week	38,582 (26%)	184 (20%)	301 (28%)	78 (18%)	45 (21%)	470 (18%)	123 (18%)	42 (28%)	39,825 (26%)
3‐4 Times per week	41,091 (28%)	92 (10%)	152 (14%)	45 (10%)	38 (18%)	320 (12%)	78 (11%)	24 (16%)	41,840 (27%)
Daily	39,630 (27%)	72 (8%)	154 (14%)	44 (10%)	30 (14%)	278 (11%)	83 (12%)	17 (11%)	40,308 (26%)

*Note:* The *p* values for comparison of each ancestry with Europeans are provided in Table S2. E: European, A: African, BFP: whole body fat percentage, HDL: high‐density lipoprotein cholesterol, MET: summed metabolic equivalent task minutes per week for all activity. The values in the brackets except for percentages are SDs.

Abbreviations: AA: African‐American, EA: East Asian: API: Asian Pacific Islander, SA: South Asian, LA‐1: Latin‐American 1, LA‐2: Latin‐American‐2, TFP: trunk fat percentage, LFP: leg fat percentage, WHR: waist–hip ratio, Townsend: Townsend deprivation index, NPX: normalized protein expression.

^∗^Individuals on lipid‐lowering medications were excluded.

**TABLE 2:** Female characteristics at baseline.

Ancestry	E	A	AA	EA	API	SA	LA‐1	LA‐2	All
*N*	185,935	1128	1735	941	356	2513	705	261	193,574
Age (years)	56.3 (8.0)	50.9 (7.5)	51.1 (7.6)	52.1 (7.5)	51.0 (7.6)	52.4 (8.2)	51.3 (7.7)	53.2 (7.7)	56.1 (8.0)
BFP	36.2 (6.8)	40.2 (6.4)	38.1 (6.9)	29.4 (6.1)	33.6 (6.4)	37.2 (6.1)	36.5 (6.9)	35.5 (6.1)	36.2 (6.8)
TFP	33.7 (7.7)	37.6 (7.3)	35.6 (7.9)	25.9 (7.2)	30.2 (7.5)	34.5 (7.3)	33.8 (7.9)	32.1 (7.2)	33.7 (7.7)
LFP	40.0 (5.4)	43.4 (5.2)	41.6 (5.6)	34.6 (4.9)	38.3 (5)	41.2 (4.6)	40.4 (5.3)	40.2 (4.9)	40.0 (5.5)
WHR	0.81 (0.07)	0.84 (0.07)	0.83 (0.07)	0.81 (0.07)	0.83 (0.07)	0.84 (0.07)	0.82 (0.07)	0.83 (0.07)	0.81 (0.07)
Leptin (NXP)									
*N*	19,130	182	219	98	36	240	103	23	20,031
Mean (SD)	0.48 (1.07)	1.3 (0.748)	0.9 (1.05)	−0.42 (0.97)	0.25 (1.05)	0.94 (0.85)	0.75 (0.95)	0.78 (0.99)	0.49 (1.07)
Glucose (mmol/L)	4.92 (0.55)	4.78 (0.55)	4.79 (0.52)	4.96 (0.55)	4.97 (0.56)	4.88 (0.53)	4.87 (0.53)	4.94 (0.48)	4.92 (0.55)
HbA1c (mmol/L)	34.8 (3.5)	36.7 (4.8)	36.2 (4.5)	35.8 (3.8)	35.8 (3.7)	36.4 (3.9)	35.0 (3.7)	35.5 (3.6)	34.8 (3.6)
Triglycerides (mmol/L)^∗^									
*N*	170,514	1043	1628	887	334	2277	669	246	177,598
Mean (SD)	1.49 (0.80)	0.97 (0.50)	1.10 (0.58)	1.50 (0.92)	1.59 (0.86)	1.63 (0.91)	1.34 (0.70)	1.58 (0.84)	1.48 (0.80)
HDL (mmol/L)^∗^									
*N*	170,514	1043	1628	887	334	2277	669	246	177,598
Mean (SD)	1.63 (0.37)	1.54 (0.34)	1.58 (0.37)	1.62 (0.37)	1.52 (0.35)	1.43 (0.33)	1.56 (0.38)	1.56 (0.36)	1.62 (0.37)
Apolipoprotein B (g/L)^∗^									
*N*	169,943	1030	1618	881	331	2269	667	245	176,984
Mean (SD)	1.06 (0.23)	0.94 (0.22)	0.98 (0.22)	0.98 (0.21)	1.00 (0.23)	1.03 (0.21)	1.02 (0.22)	1.06 (0.23)	1.05 (0.23)
Free fatty acids (mmol/L)^∗^									
*N*	95,518	520	846	458	180	1128	367	127	99,144
Mean (SD)	12.25 (2.32)	10.23 (1.84)	10.59 (1.91)	11.8 (2.27)	11.81 (2.21)	11.92 (2.35)	11.58 (2.12)	12.60 (2.47)	12.22 (2.32)
Menopause	115,698 (62%)	477 (42%)	677 (40%)	444 (47%)	141 (40%)	1256 (50%)	305 (43%)	153 (59%)	119,150 (62%)
Townsend	−1.55 (2.91)	2.97 (3.4)	2.02 (3.29)	−0.18 (3.47)	0.16 (3.29)	−0.08 (3.05)	0.72 (3.56)	1.26 (3.90)	−1.45 (2.97)
MET	2558 (2448)	2445 (2391)	2521 (2474)	2430 (2501)	2666 (2408)	2289 (2346)	2548 (2644)	3050 (2897)	2554 (2449)
Smoking status									
Never	109,247 (59%)	948 (84%)	1163 (67%)	766 (81%)	252 (71%)	2246 (89%)	403 (57%)	145 (56%)	115,170 (59%)
Previous	59,629 (32%)	106 (9%)	312 (18%)	119 (13%)	55 (15%)	138 (5%)	180 (26%)	90 (34%)	60,629 (31%)
Current	16,323 (9%)	62 (5%)	239 (14%)	41 (4%)	35 (10%)	86 (3%)	111 (16%)	22 (8%)	16,919 (9%)
Alcohol intake									
Never	13,796 (7%)	379 (34%)	268 (15%)	245 (26%)	87 (24%)	1253 (50%)	194 (28%)	32 (12%)	16,254 (8%)
Special occasions	24,858 (13%)	400 (35%)	521 (30%)	347 (37%)	129 (36%)	526 (21%)	137 (19%)	97 (37%)	27,015 (14%)
1‐3 Times per month	24,284 (13%)	117 (10%)	295 (17%)	102 (11%)	29 (8%)	207 (8%)	103 (15%)	34 (13%)	25,171 (13%)
1‐2 Times per week	49,705 (27%)	138 (12%)	360 (21%)	122 (13%)	52 (15%)	281 (11%)	124 (18%)	49 (19%)	50,831 (26%)
3‐4 Times per week	40,877 (22%)	55 (5%)	162 (9%)	59 (6%)	29 (8%)	121 (5%)	74 (10%)	22 (8%)	41,399 (21%)
Daily	32,195 (17%)	27 (2%)	109 (6%)	53 (6%)	17 (5%)	81 (3%)	68 (10%)	24 (9%)	32,574 (17%)

*Note:* The *p* values for comparison of each ancestry with Europeans are provided in Table S3. E: European, A: African, BFP: whole body fat percentage, HDL: high‐density lipoprotein cholesterol, MET: summed metabolic equivalent task minutes per week for all activity, Townsend: Townsend deprivation index. The values in the brackets except for percentages are SDs.

Abbreviations: AA: African‐American, EA: East Asian: API: Asian Pacific Islander, SA: South Asian, LA‐1: Latin‐American 1, LA‐2: Latin‐American‐2, TFP: trunk fat percentage, LFP: leg fat percentage, WHR: waist–hip ratio, NPX: normalized protein expression.

^∗^Individuals on lipid‐lowering medications were excluded.

Individuals who had undergone DXA assessment were slightly younger and included more males compared to those who had not (Table S4). Bioimpedance‐derived adiposity measures were correlated with similar DXA‐derived measures: ICC for BFP, TFP and LFP was 0.93, 0.82 and 0.89, respectively (Table S5 and Figure S6).

### 3.1. Males

#### 3.1.1. Anthropometric and Body Composition Measures

**FIGURE 1:**
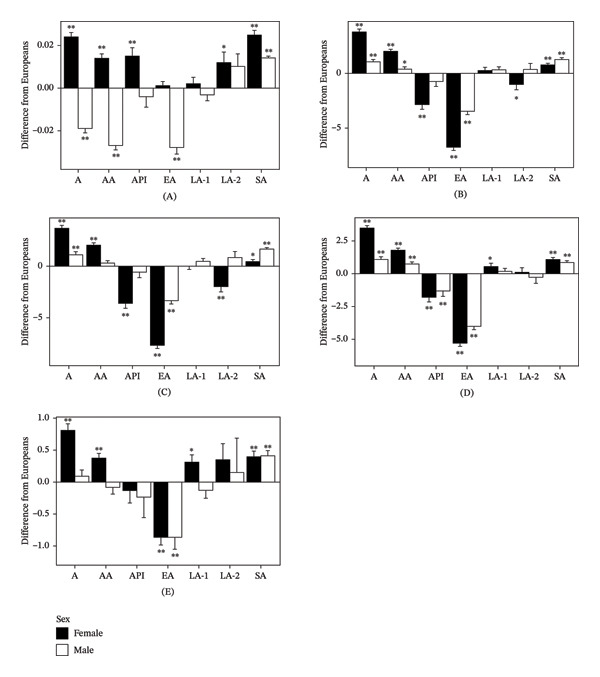
Differences in anthropometric and body composition measures in different ancestry groups compared to Europeans by sex. (A) WHR; (B) whole body fat percentage; (C) trunk fat percentage; (D) leg fat percentage; and (E) leptin. A: African, AA: African‐American, EA: East Asian: API: Asian Pacific Islander, SA: South Asian, LA‐1: Latin‐American 1, LA‐2: Latin‐American‐2, WHR: waist–hip ratio. ^∗^ *p* < 0.05 and ^∗∗^ *p* < 3.57 × 10^−3^.

WHR was significantly lower in A (*β* = −0.019 ± 0.002 (SE), *p* = 1.58 × 10^−15^), AA (*β* = −0.027 ± 0.002, *p* = 2 × 10^−16^) and EA (*β* = −0.028 ± 0.003, *p* < 2 × 10^−16^) ancestries and significantly higher in SA (*β* = 0.014 ± 0.001, *p* < 2 × 10^−16^) ancestry compared to Es. There were no significant differences in WHR between API, LA‐1 and LA‐2 ancestries compared to Es (Figure 1 and Tables S6 and S7).

BFP was significantly higher in A (*β* = 1.02 ± 0.22, *p* < 2 × 10^−16^) and SA (*β* = 1.28 ± 0.13, *p* < 2 × 10^−16^) ancestries but significantly lower in EA ancestry (*β* = −3.49 ± 0.30, *p* < 2 × 10^−16^) with no significant differences in AA, API, LA‐1 and LA‐2 ancestries compared to Es. Plasma leptin was significantly higher in SA (*β* = 0.41 ± 0.08, *p* < 4.35 × 10^−7^) and lower in EA (*β* = −0.86 ± 0.19, *p* = 3.76 × 10^−6^), with no significant difference in A ancestries compared to Es. TFP and LFP data were generally similar to BFP data. TFP was higher in A (*β* = 1.10 ± 0.25, *p* = 9.92 × 10^−6^) and SA (*β* = 1.64 ± 0.15, *p* < 2 × 10^−16^) ancestries but lower in EA ancestry (*β* = −3.37 ± 0.34, *p* < 2 × 10^−16^) compared to Es, with no significant differences for the other ancestries. LFP was higher in A (*β* = 1.08 ± 0.19, *p* = 2.20 × 10^−8^) and SA (*β* = 0.85 ± 0.11, *p* = 4.21 × 10^−14^) and lower in EA (*β* = −3.99 ± 0.27, *p* < 2 × 10^−16^) compared to Es. In addition, LFP was higher in AA (*β* = 0.71 ± 0.17, *p* = 4.04 × 10^−5^) and lower in API (*β* = −1.33 ± 0.40, *p* = 8.91 × 10^−4^) compared to the Es (Tables S6 and S7 and Figure 1).

#### 3.1.2. Glycaemic and Lipid Traits

**FIGURE 2:**
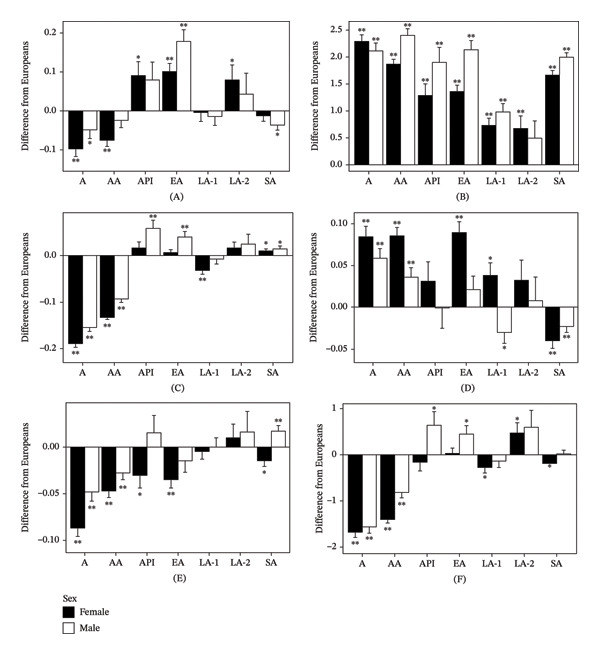
Differences in glycemic and lipid traits in different ancestry groups compared to Europeans by sex. (A) Glucose; (B) HbA1c; (C) triglycerides; (D) HDL; (E) apolipoprotein B; and (F) total free fatty acids. A: African, AA: African‐American, EA: East Asian: API: Asian Pacific Islander, SA: South Asian, LA‐1: Latin‐American 1, LA‐2: Latin‐American‐2. ^∗^ *p* < 0.05 and ^∗∗^ *p* < 3.57 × 10^−3^.

HbA1c was significantly higher in all groups except for LA‐2 compared to Es (A: *β* = 2.12 ± 0.14, *p* < 2 × 10^−16^, AA: *β* = 2.40 ± 0.12, *p* < 2 × 10^−16^, API: *β* = 1.90 ± 0.28, *p* = 8.70 × 10^−12^, EA: *β* = 2.13 ± 0.19, *p* < 2 × 10^−16^, SA: *β* = 2.00 ± 0.08, *p* < 2 × 10^−16^, LA‐1: *β* = 0.99 ± 0.15, *p* = 2.42 × 10^−11^) (Tables S8–S10 and Figure 2). The interaction between ancestry and WHR for HbA1c was nominally significant for EA (*β* = 6.42 ± 3.22, *p* = 0.046) and SA (*β* = 3.05 ± 1.33, *p* = 0.02) (Table S10). Random glucose was only higher in EA (*β* = 0.18 ± 0.03, *p* = 5.24 × 10^−9^) compared to Es (Tables S8 and S9 and Figure 2).

TG and apoB were significantly reduced in A (TG: *β* = −0.155 ± 0.009, *p* < 2 × 10^−16^, apoB: *β* = −0.048 ± 0.009, *p* = 1.78 × 10^−7^) and AA (TG: *β* = −0.093 ± 0.008, *p* < 2 × 10^−16^, apoB: *β* = −0.028 ± 0.008, *p* = 9.79 × 10^−4^) compared to Es. In SA ancestry, apoB was higher compared to Es (*β* = 0.017 ± 0.006, *p* = 2.79 × 10^−3^) with no significant difference in TG; whereas, both EA (TG: *β* = 0.040 ± 0.012, *p* = 1.02 × 10^−3^) and API (*β* = 0.058 ± 0.018, *p* = 1.73 × 10^−3^) ancestries had higher TG, with no significant differences in apoB compared to Es. TG and apoB were not significantly different in LA‐1 and LA‐2 ancestries compared to Es (Tables S8 and S9 and Figure 2).

HDL was higher in A (*β* = 0.058 ± 0.012, *p* = 8.37 × 10^−7^) and AA (*β* = 0.036 ± 0.011, *p* = 6.13 × 10^−4^) ancestries and lower in SA ancestry (*β* = −0.023 ± 0.007, *p* = 1.58 × 10^−3^) compared to Es. No differences in HDL were seen in other ancestries compared to Es (Tables S8 and S9 and Figure 2).

Total FFA was reduced in A (*β* = −1.56 ± 0.14, *p* < 2 × 10^−16^) and AA (*β* = −0.81 ± 0.12, *p* = 2.81 × 10^−11^) ancestries compared to Es. There was no significant difference in total FFA in other ancestries including SA compared to Es (Tables S8 and S9 and Figure 2).

### 3.2. Females

#### 3.2.1. Anthropometric and Body Composition Measures

WHR was significantly higher in A (*β* = 0.24 ± 0.002, *p* < 2 × 10^−16^), AA (*β* = 0.014 ± 0.002, *p* = 2.64 × 10^−14^), API (*β* = 0.015 ± 0.004, *p* = 2.2.32 × 10^−4^) and SA (*β* = 0.025 ± 0.002, *p* < 2 × 10^−16^) ancestries compared to Es. There were no differences in WHR in EA and LA ancestries compared to Es (Tables S11 and S12 and Figure 1).

BFP was significantly higher in A (*β* = 3.77 ± 0.24, *p* < 2 × 10^−16^), AA (*β* = 1.99 ± 0.19, *p* = *p* < 2 × 10^−16^) and SA (*β* = 0.74 ± 0.16, *p* = 7.62 × 10^−6^) and significantly lower in EA (*β* = −6.75 ± 0.25, *p* < 2 × 10^−16^) and API (*β* = −2.84 ± 0.42, *p* = 1.97 × 10^−11^) ancestries with no significant difference in LA ancestry compared to Es. Plasma leptin was significantly higher in A (*β* = 0.81 ± 0.10, *p* < 2 × 10^−16^), AA (*β* = 0.37 ± 0.08, *p* = 1.44 × 10^−5^) and SA (*β* = 0.40 ± 0.08, *p* = 2.47 × 10^−6^) and lower in EA (*β* = −0.86 ± 0.13, *p* = 1.58 × 10^−11^), with no significant difference in API ancestries compared to Es. TFP and LFP data were generally similar to BFP data. TFP was increased in A (*β* = 3.65 ± 0.28, *p* < 2 × 10^−16^), AA (*β* = 2.00 ± 0.22, *p* < 2 × 10^−16^) but decreased in EA (*β* = −7.75 ± 0.29, *p* < 2 × 10^−16^) and API (*β* = −3.65 ± 0.48, *p* = 5.25 × 10^−14^). However, TFP was not significantly increased in SA. Additionally, TFP was lower in LA‐2 compared to Es (*β* = −1.99 ± 0.53, *p* = 1.49 × 10^−4^). LFP was increased in A (*β* = 3.47 ± 0.19, *p* < 2 × 10^−16^), AA (*β* = 1.79 ± 0.15, *p* < 2 × 10^−16^) and SA (*β* = 1.09 ± 0.13, *p* < 2 × 10^−16^) but decreased in EA (*β* = −5.32 ± 0.20, *p* < 2 × 10^−16^) and API (*β* = −1.81 ± 0.34, *p* = 8.02 × 10^−8^) ancestries, with no significant difference in LA ancestry compared to Es (Tables S11 and S12 and Figure 1).

#### 3.2.2. Glycaemic and Lipid Traits

HbA1c was significantly increased in all ancestries compared to Es (A: *β* = 2.29 ± 0.12, *p* < 2 × 10^−16^, AA: *β* = 1.87 ± 0.09, *p* < 2 × 10^−16^, API: *β* = 1.29 ± 0.21, *p* = 4.00 × 10^−10^, EA: *β* = 1.36 ± 0.12, *p* < 2 × 10^−16^, SA: *β* = 1.66 ± 0.08, *p* < 2 × 10^−16^, LA‐1: *β* = 0.73 ± 0.14, *p* = 3.06 × 10^−7^, LA‐2: *β* = 0.68 ± 0.23, *p* = 2.63 × 10^−3^) (Tables S13–S15 and Figure 2). The interaction between ancestry and WHR for HbA1c was significant in AA (*β* = 4.72 ± 1.38, *p* = 6.3 × 10^−4^) and nominally significant in SA (*β* = 3.06 ± 1.13, *p* = 6.9 × 10^−3^) and A (*β* = 3.32 ± 1.66, *p* = 0.045) (Table S15). Random glucose was lower in A (*β* = −0.096 ± 0.020, *p* = 1.57 × 10^−6^) and AA (*β* = −0.076 ± 0.015, *p* = 9.19 × 10^−7^), and higher in EA (*β* = 0.102 ± 0.020, *p* = 6.78 × 10^−7^) with no significant differences in other ancestries compared to Es (Tables S13 and S14 and Figure 2).

TG and apoB was significantly reduced in A (TG: *β* = −0.190 ± 0.007, *p* < 2 × 10^−16^, apoB: *β* = −0.087 ± 0.008, *p* < 2 × 10^−6^) and AA (TG: *β* = −0.133 ± 0.005, *p* < 2 × 10^−16^, apoB: *β* = −0.047 ± 0.006, *p* = 1.34 × 10^−13^) compared to Es, while TG and apoB differences were not significantly different from Es in SA ancestry. EA ancestry had lower apoB (*β* = −0.035 ± 0.008, *p* = 2.74 × 10^−5^), with no significant differences in TG compared to Es. TG levels were lower (*β* = −0.032 ± 0.008, *p* = 1.01 × 10^−4^), with no significant difference in apoB in LA‐1 ancestry compared to Es (Tables S13 and S14 and Figure 2).

HDL was higher in A (*b* = 0.084 ± 0.013, *p* = 7.83 × 10^−11^), AA (*b* = 0.085 ± 0.010, *p* < 2 × 10^−16^) and EA (*b* = 0.089 ± 0.013, *p* = 1.38 × 10^−11^) ancestries and lower in SA ancestry (*b* = −0.040 ± 0.009, *p* = 5.08 × 10^−6^) compared to Es. No significant changes in HDL were seen in other ancestries compared to Es (Tables S13 and S14 and Figure 2).

Total FFA was reduced in A (*β* = −1.67 ± 0.11, *p* < 2 × 10^−16^) and AA (*β* = −1.40 ± 0.09, *p* < 2 × 10^−16^). There were no significant differences in total FFA between other ancestries and Es (Tables S13 and S14 and Figure 2).

### 3.3. Effect of Ancestry vs. Sex

#### 3.3.1. Anthropometric and Body Composition Measures

Sex generally had a larger effect on WHR, BFP, TFP, LFP and leptin compared to ancestry. However, among ancestries, the effect of EA ancestry was often larger than that observed for other ancestries. For example, the effect of EA ancestry on TFP was nearly as large as that of sex (−6.30 vs. −6.35) (Table 3).

**TABLE 3:** Effects of ancestry and sex on anthropometric and body composition measures.

	WHR			BFP			TFP			LFP			Leptin		
	Beta	SE	P	Beta	SE	P	Beta	SE	P	Beta	SE	P	Beta	SE	P
A	0.004	0.002	1.97E − 2	2.47	0.16	< 2E − 16	2.44	0.19	< 2E − 16	2.34	0.14	< 2E − 16	0.44	0.07	5.03E − 10
AA	−0.002	0.001	0.22	1.41	0.14	< 2E − 16	1.37	0.16	< 2E − 16	1.39	0.11	< 2E − 16	0.19	0.07	4.79E − 3
API	0.008	0.003	1.02E − 2	−2.04	0.31	4.07E − 11	−2.49	0.36	2.39E − 12	−1.64	0.26	2.08E − 10	−0.17	0.17	0.33
EA	−0.008	0.002	2.25E − 5	−5.67	0.19	< 2E − 16	−6.30	0.22	< 2E − 16	−4.88	0.16	< 2E − 16	−0.86	0.11	6.20E − 16
LA‐1	−0.001	0.002	0.71	0.21	0.19	0.28	0.12	0.22	0.56	0.29	0.16	0.07	0.10	0.09	0.27
LA‐2	0.012	0.004	8.74E − 4	−0.52	0.34	0.13	−1.01	0.39	1.01E − 2	−0.01	0.29	0.97	0.32	0.23	0.16
SA	0.019	0.001	< 2E − 16	0.97	0.10	< 2E − 16	1.03	0.12	< 2E − 16	0.91	0.09	< 2E − 16	0.40	0.06	7.05E − 12
Sex (male)	0.117	0.000	< 2E − 16	−11.08	0.02	< 2E − 16	−6.35	0.03	< 2E − 16	−18.17	0.02	< 2E − 16	−1.38	0.01	< 2E − 16

*Note:* A: African. Linear regression was used to test association of each ancestry vs. Europeans and sex (male) with each anthropometric and body composition measure with age, alcohol intake, Townsend deprivation index and summed metabolic equivalent task (MET) minutes per week for all activity as covariates in the model.

Abbreviations: AA: African‐American, EA: East Asian: API: Asian Pacific Islander, SA: South Asian, LA‐1: Latin‐American 1, LA‐2: Latin‐American‐2, WHR: waist–hip ratio, BFP: whole body fat percentage, TFP: trunk fat percentage, LFP: leg fat percentage.

#### 3.3.2. Glycaemic and Lipid Traits

The effects of ancestries on glycaemic and lipid traits were often greater than sex. The effect of EA ancestry on random glucose was greater than sex (0.126 vs. −0.086); the effects of A (2.23), AA (2.04), API (1.50), EA (1.60), SA (1.86) and LA‐1 (0.86) ancestries on HbA1c was greater than sex (−0.84); effects of A (−0.174), AA (−0.121), API (0.030) and LA‐1 (−0.023) ancestries on TG were greater than sex (−0.017); effect of A ancestry on apoB (−0.064) was greater than sex (−0.053); and effects of A (−1.63) and AA(−1.22) ancestries were greater than sex (−1.11) for FFA (Table 4).

**TABLE 4:** Effects of ancestry and sex on glycaemic and lipid traits.

	Glucose			HbA1c			Triglycerides			HDL			Apolipoprotein B			Free fatty acids		
	Beta	SE	P	Beta	SE	P	Beta	SE	P	Beta	SE	P	Beta	SE	P	Beta	SE	P
A	−0.074	0.015	6.84E − 7	2.23	0.09	< 2E − 16	−0.174	0.006	< 2E − 16	0.071	0.009	9.90E − 16	−0.064	0.006	< 2E − 16	−1.63	0.09	< 2E − 16
AA	−0.056	0.012	4.71E − 6	2.04	0.07	< 2E − 16	−0.121	0.005	< 2E − 16	0.060	0.007	< 2E − 16	−0.042	0.005	5.66E − 16	−1.22	0.07	< 2E − 16
API	0.087	0.028	1.70E − 3	1.50	0.17	< 2E − 16	0.030	0.010	4.52E − 3	0.016	0.017	0.34	−0.015	0.012	0.19	0.10	0.16	0.55
EA	0.126	0.017	2.22E − 13	1.60	0.10	< 2E − 16	0.014	0.006	3.04E − 2	0.065	0.010	2.04E − 10	−0.031	0.007	1.24E − 5	0.13	0.10	0.20
SA	−0.024	0.009	0.01	1.86	0.06	< 2E − 16	0.011	0.004	1.59E − 3	−0.027	0.006	2.19E − 6	0.000	0.004	0.90	−0.08	0.06	0.15
LA‐1	−0.010	0.017	0.55	0.86	0.10	< 2E − 16	−0.023	0.006	4.18E − 4	0.009	0.010	0.37	−0.003	0.007	0.68	−0.21	0.10	3.10E − 2
LA‐2	0.069	0.031	2.56E − 2	0.65	0.19	5.40E − 4	0.020	0.012	0.09	0.025	0.018	0.18	0.015	0.013	0.26	0.51	0.19	7.44E − 3
Sex (male)	−0.086	0.003	< 2E − 16	−0.84	0.02	< 2E − 16	−0.017	0.001	< 2E − 16	−0.175	0.002	< 2E − 16	−0.053	0.001	< 2E − 16	−1.11	0.02	< 2E − 16

*Note:* A: African, HDL: high‐density lipoprotein cholesterol. Linear regression was used to test association of each ancestry vs. Europeans and sex (male) with each glycaemic and lipid measure with age, fasting hours (> 3 h vs. ≤ 3 h), blood sampling hour, smoking status, alcohol intake, Townsend deprivation index and summed metabolic equivalent task (MET) minutes per week for all activity as covariates in the model.

Abbreviations: AA: African‐American, EA: East Asian: API: Asian Pacific Islander, SA: South Asian, LA‐1: Latin‐American 1, LA‐2: Latin‐American‐2.

## 4. Conclusions

Central adiposity and/or reduced leg fat is postulated to promote IR, dysglycemia and dyslipidemia in part via increased lipid flux and ectopic lipid deposition in metabolically relevant tissues [3]. Our current data raise the possibility that this paradigm, which has been consistently seen in people of E ancestry (based on self‐report)/ancestry (based on genetic principal components), might have variable contributions across ancestries, supporting the need for future confirmatory studies.

Dysglycemia: Amongst males and females, all ancestry groups except LA‐2 males (with the caveat of small sample size) had significantly higher HbA1c compared to Es even after adjustment for WHR. Notably, in unadjusted analyses increased HbA1c was seen with decreased WHR in some groups (A, AA and EA males) or no difference in WHR (API and LA‐1 males and females of EA and LA‐1 ancestries) compared to sex‐matched Es. Based on these data, we hypothesize that dysglycemia in these ancestries may be partly independent of increased WHR: this awaits confirmation with mechanistic studies.

### 4.1. Reduced Leg Fat and Adverse Metabolic Profile in People of EA and API Ancestries

Increased HbA1c was associated with reduced LFP in EA and API males and females but not other groups compared to sex‐matched Es. Dysglycemia is typically associated with dyslipidemia characterized by increased TG and TG‐rich apoB‐containing lipoproteins and reduced HDL. Dyslipidemia may be due to increased total FFA in the setting of reduced adipose storage capacity and reduced leg fat [1, 3, 4], but this warrants confirmatory studies. Reduced LFP was also seen in EA and API females but with no evidence of dyslipidemia or increased total FFA. This raises the plausibility of factors other than lipid flux contributing to the increased HbA1c seen in these groups.

### 4.2. Discordance Between Glycaemia and Lipid Parameters in A, AA, LA‐1 and LA‐2 Ancestries

Increased glycaemia is usually associated with dyslipidemia and increased lipid flux, but among LA‐1 (males and females) and LA‐2 females compared to sex‐matched Es, we did not see evidence for increased TG/apoB, low HDL or increased FFA after adjusting for WHR, suggesting other potential factors might contribute to dysglycemia.

Consistent with prior data, we report that increased glycaemia in A and AA males and females is paradoxically associated with reduced TG and apoB with increased HDL [11]. We further confirm that this also associates with reduced total FFA, suggesting that decreased lipid flux may potentially underlie this phenotype. The decrease was seen even after correcting for BFP. In a previous study comparing females based on self‐reported ethnicity, in comparison to Es, AA women with obesity had reduced adipose tissue lipolysis and plasma FFA (suggestive of reduced lipid flux) as well as VLDL–apoB100 secretion in the fasted state [20]. Although surrogate measures of IR such as HOMA‐IR are increased in AA individuals without Type 2 diabetes, clamp studies indicate a phenotype of hyperinsulinemia with no increase in IR. It has been postulated that this may be an early driver of Type 2 diabetes, which initiates IR and beta cell dysfunction [21, 22]. Confirmatory studies are needed to assess whether hyperinsulinemia relative to insulin sensitivity could potentially decrease adipose tissue lipolysis, total FFA and protect against dyslipidemia. The protection against dyslipidemia has been invoked as a potential contributor to reduced coronary artery disease risk in A and AA individuals [23].

### 4.3. Dysglycemia in SA Individuals

SA males and females had higher WHR and but increased dysglycemia relative to E ancestry even after adjusting for WHR. Prior reports postulate that this phenotype in conjunction with dyslipidemia contributes to the high cardiovascular risk amongst SA individuals [12, 23, 24]. Intriguingly, with the exception of a significant reduction in HDL in SA females, we did not detect any significant lipid abnormalities. A caveat to interpreting this data is the relatively small sample size, as plasma TG in both sexes was nominally higher. The largest meta‐analyses of body fat composition in SA individuals (based on self‐report) indicates higher liver fat content in the setting of higher subcutaneous but not visceral fat compared to Es [25]. Our finding of no significant increase in plasma FFA in SA suggests the possibility of factors other than altered lipid flux might contribute to both increased hepatic lipid in SA as well as dysglycemia, but this awaits formal confirmation. Recent cluster‐based portioned genetic risk scores in SAs with Type 2 diabetes in the United Kingdom indicate that multiple pathways contribute to the aetiology of Type 2 diabetes. Overall portioned genetic risk scores for beta cell function 1 and 2 clusters showed the strongest association with Type 2 diabetes, underscoring the likely role of beta cell dysfunction in dysglycemia and Type 2 diabetes in SA [26].

### 4.4. Strengths and Limitations

The strengths of this study include the large well‐phenotyped cohort and standardized methods for data collection and analyses. Furthermore, we undertook sex‐stratified analyses. Because of differences in allele frequencies and linkage disequilibrium across ancestral groups, analyses in subgroups defined by genetic principal components will facilitate further studies investigating underlying biological mechanisms.

Limitations of the study include small sample sizes for some groups and lack of availability for some biochemical measures such as leptin, apoB and total FFA for all participants; furthermore, not all measures were undertaken in the fasted state. Although bioimpedance correlates well with DXA‐derived fat mass, it does not differentiate subcutaneous and visceral abdominal fat from total trunk fat. Furthermore, we did not assess the independent association of each fat depot with glycaemic and lipid traits. As this is a cross‐sectional observational study, it is prone to confounding and bias, precluding causal inference to the associations seen. We elected to study patients without diabetes and/or those on lipid‐lowering medications but cannot exclude the possibility that other conditions or medications may have influenced outcomes. We did not assess to what extent dietary and other social factors contributed to these differences. Furthermore, the demographics of participants in the UK Biobank are not representative of the general population, with under‐representation of non‐E individuals [27].

### 4.5. Conclusions

In conclusion, our data suggest that factors beyond fat distribution and increased lipid flux could potentially contribute to glycaemic and lipid traits across ancestries compared to Es and that dysglycemia does not necessarily associate with dyslipidemia and increased fatty acids across ancestries. The clinical implications of these findings warrant further exploration, but notably, current guidelines recommend using measures of central adiposity such as WHR in addition to body mass index to screen and treat for obesity and metabolic disease, and diagnostic criteria for metabolic syndrome implicitly assume that dysglycemia and dyslipidemia have shared associations [28–30]. Further mechanistic studies are needed to understand the reason(s) for these differing phenotypes.

## Author Contributions

Delnaz Roshandel analysed the data. Satya Dash and Delnaz Roshandel wrote the manuscript. All authors designed the study and edited the manuscript. Andrew D. Paterson and Satya Dash are the guarantors of this work and, as such, had full access to all study data available through UK Biobank Research Analysis Platform (RAP) and take responsibility for the integrity of the data and the accuracy of the data analysis.

## Funding

The study was funded by a project grant from CIHR (PJT 450973, Satya Dash) and operating grant from Heart & Stroke Foundation (G‐ ‐21‐0031489, Satya Dash).

## Conflicts of Interest

The authors declare no conflicts of interest.

## Supporting Information

Additional supporting information can be found online in the Supporting Information section.

## Supporting information


**Supporting Information** Supporting File: Supporting Tables S1–9. Supporting Figures S1–6. Strobe checklist.

## Data Availability

The data that support the findings of this study are available in the supporting information of this article. UK Biobank data are available for use by eligible researchers from academic, charity, government and commercial organisations across the world for health‐related research that is in the public interest (https://www.ukbiobank.ac.uk/use-our-data/apply-for-access/).
