# Supplementary material for: Anthropometric and Metabolic Traits Across Ancestries in the UK Biobank
Source: J Obes. 2026 Jul 21;2026:5149353. doi: 10.1155/jobe/5149353 (PMC13387133; doi:10.1155/jobe/5149353)
Supplement: Supplementary file 1 — Supporting Information Supporting File: Supporting Tables S1–9. Supporting Figures S1–6. Strobe checklist. [file JOBE-2026-5149353-s001.docx]

**Supplementary**

**Supplementary Tables**

**Table S1:** Individuals included in the analysis after each exclusion step by ancestry

| **Ancestry** | **E**  **N (%)** | **A**  **N (%)** | **AA**  **N (%)** | **EA**  **N (%)** | **API**  **N (%)** | **SA**  **N (%)** | **LA-1**  **N (%)** | **LA-2**  **N (%)** | **All**  **N (%)** |
| --- | --- | --- | --- | --- | --- | --- | --- | --- | --- |
| **Initial** | 460,639 | 4,211 | 4,965 | 1,953 | 902 | 9,546 | 2,145 | 574 | 484,935 |
| **Individuals with Diabetes** | 48,732 (11) | 994 (24) | 1,070 (22) | 254 (13) | 157 (17) | 3,046 (32) | 339 (16) | 60 (10) | 54,652 (11) |
| **Missing Covariates** | 78,442 (17) | 1,177 (28) | 1,087 (22) | 325 (17) | 175 (19) | 1,379 (14) | 406 (19) | 105 (11) | 83,096 (17) |
| **Included in the Analysis** | 333,465 (72) | 2,040 (48) | 2,808 (57) | 1,374 (70) | 570 (63) | 5,121 (54) | 1,400 (65) | 409 (71) | 347,187 (72) |
| **On Lipid Lowering Medications** | 38,168 (11) | 149 (7) | 180 (6) | 83 (6) | 41 (7) | 685 (13) | 125 (9) | 31 (8) | 39,462 (11) |
| **Included in Lipid Analysis** | 295,297 (89) | 1,891 (93) | 2,628 (94) | 1,291 (94) | 529 (93) | 4,436 (87) | 1,275 (91) | 378 (92) | 307,725 (89) |

E: European, A: African, AA: African-American, EA: East Asian: API: Asian Pacific Islander, SA: South Asian, LA-1: Latin-American 1, LA-2: Latin-American-2

The percentages are all column percentages. For “On lipid lowering medications” and “Included in lipid analysis”, the denominator is “Included in the analysis”.

**Table S2:** The comparison of male characteristics in each ancestry vs. Europeans

| **Ancestry** | **A** | **AA** | **EA** | **API** | **SA** | **LA-1** | **LA-2** |
| --- | --- | --- | --- | --- | --- | --- | --- |
| **Age (Years)** | <2E-16 | <2E-16 | <2E-16 | <2E-16 | <2E-16 | <2E-16 | 5.62E-12 |
| **Townsend** | <2E-16 | <2E-16 | <2E-16 | <2E-16 | <2E-16 | <2E-16 | <2E-16 |
| **MET** | 6.83E-3 | 0.68 | 1.11E-4 | 6.98E-3 | <2E-16 | 3.24E-3 | 0.86 |
| **Smoking Status** | <2E-16 | 2.16E-8 | 2.58E-6 | 0.24 | <2E-16 | 6.87E-14 | 3.89E-5 |
| **Alcohol Intake** | <2E-16 | <2E-16 | <2E-16 | <2E-16 | <2E-16 | <2E-16 | 2.74E-11 |

All values in the cells are p-value. The sample size, mean (SE) and N (%) are provided in Table 1.

A: African, AA: African-American, EA: East Asian: API: Asian Pacific Islander, SA: South Asian, LA-1: Latin-American 1, LA-2: Latin-American-2, MET: summed MET (Metabolic Equivalent Task) minutes per week for all activity, Townsend: Townsend deprivation index

Baseline characteristics were compared between each ethnic group and Europeans using t-test for age; Wilcoxon rank-sum test for Townsend and MET; and Cochran-Armitage trend test for smoking status and alcohol intake.

**Table S3:** The comparison of female characteristics in each ancestry vs. Europeans

| **Ancestry** | **A** | **AA** | **EA** | **API** | **SA** | **LA-1** | **LA-2** |
| --- | --- | --- | --- | --- | --- | --- | --- |
| **Age (Years)** | <2E-16 | <2E-16 | <2E-16 | <2E-16 | <2E-16 | <2E-16 | 6.07E-10 |
| **Townsend** | <2E-16 | <2E-16 | <2E-16 | <2E-16 | <2E-16 | <2E-16 | <2E-16 |
| **MET** | 0.12 | 0.35 | 2.54E-2 | 0.22 | 9.33E-9 | 0.45 | 2.89E-2 |
| **Smoking Status** | <2E-16 | 1.86E-2 | <2E-16 | 1.72E-4 | <2E-16 | 1.11E-3 | 0.57 |
| **Alcohol Intake** | <2E-16 | <2E-16 | <2E-16 | <2E-16 | <2E-16 | <2E-16 | <2E-16 |
| **Menopause** | <2E-16 | <2E-16 | <2E-16 | <2E-16 | <2E-16 | <2E-16 | 0.26 |

All values in the cells are p-value. The sample size, mean (SE) and N (%) are provided in Table 2.

A: African, AA: African-American, EA: East Asian: API: Asian Pacific Islander, SA: South Asian, LA-1: Latin-American 1, LA-2: Latin-American-2, MET: summed MET (Metabolic Equivalent Task) minutes per week for all activity, Townsend: Townsend deprivation index

Baseline characteristics were compared between each ethnic group and Europeans using t-test for age; Wilcoxon rank-sum test for Townsend and MET; Cochran–Armitage trend test for smoking status and alcohol intake; and chi-squared test for menopausal status.

**Table S4:** Characteristics of the individuals with and without DXA at Assessment 2

| **DXA** | **N** | **Age at recruitment (Years)**  **Mean (SD)** | **Sex (Female)**  **N (%)** |
| --- | --- | --- | --- |
| **No** | 454,513 | 56.7 (8.1) | 248,567 (54.7%) |
| **Yes** | 47,756 | 55.1 (7.6) | 24,671 (51.7%) |

The differences in sex (Chi-squared test) and age at recruitment (2-sample t-test) between individuals with and without DXA are both significant (p < 2.2e-16).

**Table S5:** Intra-Class correlation coefficient (ICC) between fat distributions measured by impedance and DXA at Assessment 2 (N = 45,203)

|  | **All** | **Males** | **Females** |
| --- | --- | --- | --- |
| **BFP** | 0.93 | 0.86 | 0.91 |
| **TFP** | 0.82 | 0.82 | 0.82 |
| **LFP** | 0.89 | 0.68 | 0.71 |

BFP: Whole body fat percentage, TFP: Trunk fat percentage, LFP: Legs fat percentage

**Table S6:** Association of ancestry with anthropometric and body composition measures compared to Europeans in males

|  | **WHR** | | | **BFP** | | | **TFP** | | | **LFP** | | | **Leptin** | | |
| --- | --- | --- | --- | --- | --- | --- | --- | --- | --- | --- | --- | --- | --- | --- | --- |
|  | **Beta** | **SE** | **P** | **Beta** | **SE** | **P** | **Beta** | **SE** | **P** | **Beta** | **SE** | **P** | **Beta** | **SE** | **P** |
| **African** | -0.019 | 0.002 | 1.58E-15 | 1.02 | 0.22 | 2.06E-6 | 1.10 | 0.25 | 9.92E-6 | 1.08 | 0.19 | 2.20E-8 | 0.09 | 0.10 | 0.40 |
| **African American** | -0.027 | 0.002 | <2E-16 | 0.38 | 0.19 | 4.94E-2 | 0.26 | 0.23 | 0.24 | 0.71 | 0.17 | 4.04E-5 | -0.08 | 0.11 | 0.46 |
| **Asian Pacific Islander** | -0.004 | 0.005 | 0.44 | -0.73 | 0.45 | 0.11 | -0.60 | 0.52 | 0.25 | -1.33 | 0.40 | 8.91E-4 | -0.24 | 0.31 | 0.44 |
| **East Asian** | -0.028 | 0.003 | <2E-16 | -3.49 | 0.30 | <2E-16 | -3.37 | 0.34 | <2E-16 | -3.99 | 0.27 | <2E-16 | -0.86 | 0.19 | 3.76E-6 |
| **Latin-American 1** | -0.003 | 0.003 | 0.32 | 0.34 | 0.24 | 0.15 | 0.45 | 0.27 | 0.10 | 0.18 | 0.21 | 0.41 | -0.13 | 0.13 | 0.32 |
| **Latin-American 2** | 0.010 | 0.006 | 0.08 | 0.38 | 0.52 | 0.46 | 0.76 | 0.60 | 0.21 | -0.26 | 0.46 | 0.57 | 0.15 | 0.54 | 0.78 |
| **South Asian** | 0.014 | 0.001 | <2E-16 | 1.28 | 0.13 | <2E-16 | 1.64 | 0.15 | <2E-16 | 0.85 | 0.11 | 4.21E-14 | 0.41 | 0.08 | 4.35E-7 |

WHR: Waist-hip ratio, BFP: Whole body fat percentage, TFP: Trunk fat percentage, LFP: Legs fat percentage

Linear regression was used to test association of ethnicity with WHR, BFP, TFP, LFP and leptin including age, smoking status, alcohol intake frequency, Townsend depravation index, and summed MET minutes per week for all activities as covariates in the model.

**Table S7:** Association of ancestry with anthropometric and body composition measures compared to Europeans in males adding centered quadratic age as a covariate in the model

|  | **WHR** | | | **BFP** | | | **TFP** | | | **LFP** | | | **Leptin** | | |
| --- | --- | --- | --- | --- | --- | --- | --- | --- | --- | --- | --- | --- | --- | --- | --- |
|  | **Beta** | **SE** | **P** | **Beta** | **SE** | **P** | **Beta** | **SE** | **P** | **Beta** | **SE** | **P** | **Beta** | **SE** | **P** |
| **African** | -0.018 | 0.002 | 7.07E-15 | 1.03 | 0.22 | 1.59E-6 | 1.12 | 0.25 | 8.01E-6 | 1.09 | 0.19 | 1.36E-8 | 0.09 | 0.10 | 0.38 |
| **African American** | -0.027 | 0.002 | <2E-16 | 0.39 | 0.19 | 4.56E-2 | 0.27 | 0.23 | 0.23 | 0.72 | 0.17 | 3.20E-5 | -0.08 | 0.11 | 0.48 |
| **Asian Pacific Islander** | -0.003 | 0.005 | 0.52 | -0.71 | 0.45 | 0.12 | -0.58 | 0.52 | 0.26 | -1.31 | 0.40 | 1.10E-3 | -0.24 | 0.31 | 0.44 |
| **East Asian** | -0.028 | 0.003 | <2E-16 | -3.49 | 0.30 | <2E-16 | -3.36 | 0.34 | <2E-16 | -3.98 | 0.27 | <2E-16 | -0.86 | 0.19 | 4.04E-6 |
| **Latin-American 1** | -0.002 | 0.003 | 0.37 | 0.35 | 0.24 | 0.14 | 0.45 | 0.27 | 0.10 | 0.18 | 0.21 | 0.39 | -0.13 | 0.13 | 0.31 |
| **Latin-American 2** | 0.010 | 0.006 | 0.07 | 0.39 | 0.52 | 0.45 | 0.77 | 0.60 | 0.20 | -0.25 | 0.46 | 0.59 | 0.15 | 0.54 | 0.78 |
| **South Asian** | 0.014 | 0.001 | <2E-16 | 1.29 | 0.13 | <2E-16 | 1.65 | 0.15 | <2E-16 | 0.86 | 0.11 | 1.60E-14 | 0.41 | 0.08 | 3.85E-7 |

WHR: Waist-hip ratio, BFP: Whole body fat percentage, TFP: Trunk fat percentage, LFP: Legs fat percentage

Linear regression was used to test association of ethnicity with WHR, BFP, TFP, LFP and leptin including age, centered quadratic age, smoking status, alcohol intake frequency, Townsend depravation index, and summed MET minutes per week for all activities as covariates in the model.

**Table S8:** Association of ancestry with glycemic and lipid traits compared to Europeans in males

|  | **Glucose** | | | **HbA1c** | | | **Triglycerides*** | | | **HDL*** | | | **Apolipoprotein B*** | | | **Total Free Fatty Acids*** | | |
| --- | --- | --- | --- | --- | --- | --- | --- | --- | --- | --- | --- | --- | --- | --- | --- | --- | --- | --- |
|  | **Beta** | **SE** | **P** | **Beta** | **SE** | **P** | **Beta** | **SE** | **P** | **Beta** | **SE** | **P** | **Beta** | **SE** | **P** | **Beta** | **SE** | **P** |
| **A** | -0.048 | 0.022 | 3.10E-2 | 2.12 | 0.14 | <2E-16 | -0.155 | 0.009 | <2E-16 | 0.058 | 0.012 | 8.37E-7 | -0.048 | 0.009 | 1.78E-7 | -1.56 | 0.14 | <2E-16 |
| **AA** | -0.024 | 0.020 | 0.24 | 2.40 | 0.12 | <2E-16 | -0.093 | 0.008 | <2E-16 | 0.036 | 0.011 | 6.13E-4 | -0.028 | 0.008 | 9.79E-4 | -0.81 | 0.12 | 2.81E-11 |
| **API** | 0.080 | 0.045 | 0.08 | 1.90 | 0.28 | 8.70E-12 | 0.058 | 0.018 | 1.73E-3 | -0.001 | 0.025 | 0.98 | 0.015 | 0.019 | 0.44 | 0.64 | 0.29 | 2.81E-2 |
| **EA** | 0.179 | 0.031 | 5.24E-9 | 2.13 | 0.19 | <2E-16 | 0.040 | 0.012 | 1.02E-3 | 0.021 | 0.016 | 0.19 | -0.015 | 0.013 | 0.25 | 0.45 | 0.19 | 1.60E-2 |
| **LA-1** | -0.013 | 0.024 | 0.60 | 0.99 | 0.15 | 2.42E-11 | -0.008 | 0.010 | 0.40 | -0.030 | 0.013 | 2.60E-2 | 0.000 | 0.010 | 0.99 | -0.13 | 0.14 | 0.37 |
| **LA-2** | 0.044 | 0.053 | 0.41 | 0.49 | 0.33 | 0.13 | 0.024 | 0.021 | 0.26 | 0.008 | 0.028 | 0.79 | 0.016 | 0.022 | 0.47 | 0.60 | 0.36 | 0.10 |
| **SA** | -0.036 | 0.013 | 5.90E-3 | 2.00 | 0.08 | <2E-16 | 0.014 | 0.005 | 8.06E-3 | -0.023 | 0.007 | 1.58E-3 | 0.017 | 0.006 | 2.75E-3 | 0.02 | 0.08 | 0.79 |

A: African, AA: African-American, EA: East Asian: API: Asian Pacific Islander, SA: South Asian, LA-1: Latin-American 1, LA-2: Latin-American-2, HDL: High-density lipoprotein cholesterol

Linear regression was used to test association of ancestry with glucose, HbA1c, triglyceride, HDL, apolipoprotein B and total free fatty acids including age, WHR, fasting hours (>3 hours vs. ≤3hours), blood sampling hour, smoking status, alcohol intake frequency, Townsend depravation index, and summed MET minutes per week for all activities as covariates in the model.

* Individuals on lipid lowering medications were excluded from the analysis.

**Table S9:** Association of ancestry with glycemic and lipid traits compared to Europeans in males adding centered quadratic age as a covariate in the model

|  | **Glucose** | | | **HbA1c** | | | **Triglycerides*** | | | **HDL*** | | | **Apolipoprotein B*** | | | **Total Free Fatty Acids*** | | |
| --- | --- | --- | --- | --- | --- | --- | --- | --- | --- | --- | --- | --- | --- | --- | --- | --- | --- | --- |
|  | **Beta** | **SE** | **P** | **Beta** | **SE** | **P** | **Beta** | **SE** | **P** | **Beta** | **SE** | **P** | **Beta** | **SE** | **P** | **Beta** | **SE** | **P** |
| **A** | -0.049 | 0.022 | 2.73E-2 | 2.13 | 0.14 | <2E-16 | -0.154 | 0.009 | <2E-16 | 0.059 | 0.012 | 4.55E-7 | -0.046 | 0.009 | 7.66E-7 | -1.54 | 0.14 | <2E-16 |
| **AA** | -0.024 | 0.020 | 0.22 | 2.40 | 0.12 | <2E-16 | -0.093 | 0.008 | <2E-16 | 0.037 | 0.011 | 4.83E-4 | -0.026 | 0.008 | 1.58E-3 | -0.80 | 0.12 | 7.02E-11 |
| **API** | 0.079 | 0.045 | 0.08 | 1.91 | 0.28 | 7.57E-12 | 0.059 | 0.018 | 1.50E-3 | 0.002 | 0.025 | 0.95 | 0.019 | 0.019 | 0.33 | 0.67 | 0.29 | 2.01E-2 |
| **EA** | 0.178 | 0.031 | 5.70E-9 | 2.13 | 0.19 | <2E-16 | 0.040 | 0.012 | 9.84E-4 | 0.021 | 0.016 | 0.18 | -0.014 | 0.013 | 0.27 | 0.45 | 0.19 | 1.53E-2 |
| **LA-1** | -0.013 | 0.024 | 0.59 | 0.99 | 0.15 | 2.22E-11 | -0.008 | 0.010 | 0.41 | -0.029 | 0.013 | 2.99E-2 | 0.001 | 0.010 | 0.91 | -0.11 | 0.14 | 0.43 |
| **LA-2** | 0.043 | 0.053 | 0.42 | 0.49 | 0.33 | 0.13 | 0.024 | 0.021 | 0.25 | 0.008 | 0.028 | 0.76 | 0.018 | 0.022 | 0.43 | 0.59 | 0.36 | 0.10 |
| **SA** | -0.037 | 0.013 | 4.52E-3 | 2.00 | 0.08 | <2E-16 | 0.015 | 0.005 | 6.16E-3 | -0.022 | 0.007 | 3.00E-3 | 0.020 | 0.006 | 5.75E-4 | 0.04 | 0.08 | 0.60 |

A: African, AA: African-American, EA: East Asian: API: Asian Pacific Islander, SA: South Asian, LA-1: Latin-American 1, LA-2: Latin-American-2, HDL: High-density lipoprotein cholesterol

Linear regression was used to test association of ancestry with glucose, HbA1c, triglyceride, HDL, apolipoprotein B and total free fatty acids including age, centered quadratic age, WHR, fasting hours (>3 hours vs. ≤3hours), blood sampling hour, smoking status, alcohol intake frequency, Townsend depravation index, and summed MET minutes per week for all activities as covariates in the model.

* Individuals on lipid lowering medications were excluded from the analysis.

**Table S10:** Interaction between ancestry and WHR affecting HbA1c in males

| **Ancestry** | **Beta** | **SE** | **P** |
| --- | --- | --- | --- |
| A | 3.19 | 2.25 | 0.16 |
| AA | -2.44 | 2.04 | 0.23 |
| API | 7.86 | 5.07 | 0.12 |
| EA | 6.42 | 3.22 | 4.58E-2 |
| LA-1 | -0.61 | 2.50 | 0.81 |
| LA-2 | -0.72 | 5.51 | 0.90 |
| SA | 3.05 | 1.33 | 2.20E-2 |

**Table S11:** Association of ancestry with anthropometric and body composition measures compared to Europeans in females

|  | **WHR** | | | **BFP** | | | **TFP** | | | **LFP** | | | **Leptin** | | |
| --- | --- | --- | --- | --- | --- | --- | --- | --- | --- | --- | --- | --- | --- | --- | --- |
|  | **Beta** | **SE** | **P** | **Beta** | **SE** | **P** | **Beta** | **SE** | **P** | **Beta** | **SE** | **P** | **Beta** | **SE** | **P** |
| **A** | 0.024 | 0.002 | <2E-16 | 3.77 | 0.24 | <2E-16 | 3.65 | 0.28 | <2E-16 | 3.47 | 0.19 | <2E-16 | 0.81 | 0.10 | <2E-16 |
| **AA** | 0.014 | 0.002 | 2.64E-14 | 1.99 | 0.19 | <2E-16 | 2.00 | 0.22 | <2E-16 | 1.79 | 0.15 | <2E-16 | 0.37 | 0.08 | 1.44E-5 |
| **API** | 0.015 | 0.004 | 2.32E-4 | -2.84 | 0.42 | 1.97E-11 | -3.65 | 0.48 | 5.25E-14 | -1.81 | 0.34 | 8.02E-8 | -0.13 | 0.21 | 0.54 |
| **EA** | 0.001 | 0.002 | 0.64 | -6.75 | 0.25 | <2E-16 | -7.75 | 0.29 | <2E-16 | -5.32 | 0.20 | <2E-16 | -0.86 | 0.13 | 1.58E-11 |
| **LA-1** | 0.002 | 0.003 | 0.52 | 0.25 | 0.29 | 0.39 | -0.03 | 0.33 | 0.92 | 0.56 | 0.23 | 1.51E-2 | 0.31 | 0.12 | 7.51E-3 |
| **LA-2** | 0.013 | 0.005 | 5.57E-3 | -1.04 | 0.46 | 2.37E-2 | -1.99 | 0.53 | 1.49E-4 | 0.10 | 0.37 | 0.78 | 0.35 | 0.25 | 0.17 |
| **SA** | 0.025 | 0.002 | <2E-16 | 0.74 | 0.16 | 7.62E-6 | 0.43 | 0.19 | 2.12E-2 | 1.09 | 0.13 | <2E-16 | 0.40 | 0.08 | 2.47E-6 |

A: African, AA: African-American, EA: East Asian: API: Asian Pacific Islander, SA: South Asian, LA-1: Latin-American 1, LA-2: Latin-American-2

WHR: Waist-hip ratio, BFP: Whole body fat percentage, TFP: Trunk fat percentage, LFP: Legs fat percentage

Linear regression was used to test association of ethnicity with WHR, BFP, TFP, LFP and leptin including age, menopause status, smoking status, alcohol intake frequency, Townsend depravation index, and summed MET minutes per week for all activities as covariates in the model.

**Table S12:** Association of ancestry with anthropometric and body composition measures compared to Europeans in females adding centered quadratic age as a covariate in the model

|  | **WHR** | | | **BFP** | | | **TFP** | | | **LFP** | | | **Leptin** | | |
| --- | --- | --- | --- | --- | --- | --- | --- | --- | --- | --- | --- | --- | --- | --- | --- |
|  | **Beta** | **SE** | **P** | **Beta** | **SE** | **P** | **Beta** | **SE** | **P** | **Beta** | **SE** | **P** | **Beta** | **SE** | **P** |
| **A** | 0.024 | 0.002 | <2E-16 | 3.79 | 0.24 | <2E-16 | 3.66 | 0.28 | <2E-16 | 3.49 | 0.19 | <2E-16 | 0.81 | 0.10 | 2.29E-16 |
| **AA** | 0.014 | 0.002 | 2.16E-14 | 2.01 | 0.19 | <2E-16 | 2.01 | 0.22 | <2E-16 | 1.80 | 0.15 | <2E-16 | 0.37 | 0.08 | 1.35E-5 |
| **API** | 0.015 | 0.004 | 2.12E-4 | -2.82 | 0.42 | 2.60E-11 | -3.63 | 0.48 | 7.05E-14 | -1.79 | 0.34 | 1.03E-7 | -0.13 | 0.21 | 0.54 |
| **EA** | 0.001 | 0.002 | 0.66 | -6.76 | 0.25 | <2E-16 | -7.76 | 0.29 | <2E-16 | -5.34 | 0.20 | <2E-16 | -0.86 | 0.13 | 1.53E-11 |
| **LA-1** | 0.002 | 0.003 | 0.50 | 0.27 | 0.29 | 0.35 | -0.01 | 0.33 | 0.97 | 0.57 | 0.23 | 1.21E-2 | 0.31 | 0.12 | 7.64E-3 |
| **LA-2** | 0.013 | 0.005 | 5.73E-3 | -1.05 | 0.46 | 2.17E-2 | -2.01 | 0.53 | 1.29E-4 | 0.09 | 0.36 | 0.81 | 0.34 | 0.25 | 0.17 |
| **SA** | 0.026 | 0.002 | <2E-16 | 0.76 | 0.16 | 3.47E-6 | 0.46 | 0.19 | 1.46E-2 | 1.11 | 0.13 | <2E-16 | 0.40 | 0.08 | 2.29E-6 |

A: African, AA: African-American, EA: East Asian: API: Asian Pacific Islander, SA: South Asian, LA-1: Latin-American 1, LA-2: Latin-American-2

WHR: Waist-hip ratio, BFP: Whole body fat percentage, TFP: Trunk fat percentage, LFP: Legs fat percentage

Linear regression was used to test association of ethnicity with WHR, BFP, TFP, LFP and leptin including age, centered quadratic age, menopause status, smoking status, alcohol intake frequency, Townsend depravation index, and summed MET minutes per week for all activities as covariates in the model.

**Table S13:** Association of ancestry with glycemic and lipid traits compared to Europeans in females

|  | **Glucose** | | | **HbA1c** | | | **Triglyceride*** | | | **HDL*** | | | **Apolipoprotein B*** | | | **Total Free Fatty Acids*** | | |
| --- | --- | --- | --- | --- | --- | --- | --- | --- | --- | --- | --- | --- | --- | --- | --- | --- | --- | --- |
|  | **Beta** | **SE** | **P** | **Beta** | **SE** | **P** | **Beta** | **SE** | **P** | **Beta** | **SE** | **P** | **Beta** | **SE** | **P** | **Beta** | **SE** | **P** |
| **A** | -0.096 | 0.020 | 1.57E-6 | 2.29 | 0.12 | <2E-16 | -0.190 | 0.007 | <2E-16 | 0.084 | 0.013 | 7.83E-11 | -0.087 | 0.008 | <2E-16 | -1.67 | 0.11 | <2E-16 |
| **AA** | -0.076 | 0.015 | 9.19E-7 | 1.87 | 0.09 | <2E-16 | -0.133 | 0.005 | <2E-16 | 0.085 | 0.010 | <2E-16 | -0.047 | 0.006 | 1.34E-13 | -1.40 | 0.09 | <2E-16 |
| **API** | 0.092 | 0.034 | 7.18E-3 | 1.29 | 0.21 | 4E-10 | 0.017 | 0.012 | 0.16 | 0.031 | 0.022 | 0.16 | -0.030 | 0.014 | 3.84E-2 | -0.16 | 0.19 | 0.42 |
| **EA** | 0.102 | 0.020 | 6.78E-7 | 1.36 | 0.12 | <2E-16 | 0.006 | 0.007 | 0.37 | 0.089 | 0.013 | 1.38E-11 | -0.035 | 0.008 | 2.74E-5 | 0.03 | 0.11 | 0.78 |
| **LA-1** | -0.003 | 0.024 | 0.91 | 0.73 | 0.14 | 3.06E-7 | -0.032 | 0.008 | 1.01E-4 | 0.038 | 0.015 | 1.23E-02 | -0.004 | 0.010 | 0.70 | -0.27 | 0.13 | 3.95E-2 |
| **LA-2** | 0.080 | 0.038 | 3.27E-2 | 0.68 | 0.23 | 2.63E-3 | 0.016 | 0.013 | 0.23 | 0.032 | 0.024 | 0.19 | 0.010 | 0.016 | 0.52 | 0.47 | 0.21 | 2.69E-2 |
| **SA** | -0.012 | 0.014 | 0.36 | 1.66 | 0.08 | <2E-16 | 0.010 | 0.005 | 3.29E-2 | -0.040 | 0.009 | 5.08E-6 | -0.015 | 0.006 | 6.08E-3 | -0.19 | 0.08 | 1.34E-2 |

A: African, AA: African-American, EA: East Asian: API: Asian Pacific Islander, SA: South Asian, LA-1: Latin-American 1, LA-2: Latin-American-2, HDL: High-density lipoprotein cholesterol

Linear regression was used to test association of ancestry with glucose, HbA1c, triglyceride, HDL, apolipoprotein B and total fatty acids including age, WHR, menopause status, fasting hours (>3 hours vs. ≤3 hours), blood sampling hour, smoking status, alcohol intake frequency, Townsend depravation index, and summed MET minutes per week for all activities as covariates in the model.

* Individuals on lipid lowering medications were excluded from the analysis.

**Table S14:** Association of ancestry with glycemic and lipid traits compared to Europeans in females adding centered quadratic age as a covariate in the model

|  | **Glucose** | | | **HbA1c** | | | **Triglyceride*** | | | **HDL*** | | | **Apolipoprotein B*** | | | **Total Free Fatty Acids*** | | |
| --- | --- | --- | --- | --- | --- | --- | --- | --- | --- | --- | --- | --- | --- | --- | --- | --- | --- | --- |
|  | **Beta** | **SE** | **P** | **Beta** | **SE** | **P** | **Beta** | **SE** | **P** | **Beta** | **SE** | **P** | **Beta** | **SE** | **P** | **Beta** | **SE** | **P** |
| **A** | -0.096 | 0.020 | 1.43E-6 | 2.30 | 0.12 | <2E-16 | -0.190 | 0.007 | <2E-16 | 0.085 | 0.013 | 4.97E-11 | -0.087 | 0.008 | <2E-16 | -1.66 | 0.11 | <2E-16 |
| **AA** | -0.076 | 0.015 | 8.33E-7 | 1.87 | 0.09 | <2E-16 | -0.133 | 0.005 | <2E-16 | 0.085 | 0.010 | <2E-16 | -0.047 | 0.006 | 2.35E-13 | -1.39 | 0.09 | <2E-16 |
| **API** | 0.092 | 0.034 | 7.56E-3 | 1.31 | 0.21 | 2.59E-10 | 0.018 | 0.012 | 0.15 | 0.033 | 0.022 | 0.14 | -0.028 | 0.014 | 4.60E-2 | -0.15 | 0.19 | 0.43 |
| **EA** | 0.102 | 0.020 | 6.21E-7 | 1.35 | 0.12 | <2E-16 | 0.006 | 0.007 | 0.40 | 0.088 | 0.013 | 2.27E-11 | -0.036 | 0.008 | 1.56E-5 | 0.02 | 0.11 | 0.86 |
| **LA-1** | -0.003 | 0.024 | 0.90 | 0.74 | 0.14 | 1.98E-7 | -0.031 | 0.008 | 1.29E-4 | 0.039 | 0.015 | 9.56E-3 | -0.003 | 0.010 | 0.79 | -0.25 | 0.13 | 4.94E-2 |
| **LA-2** | 0.081 | 0.038 | 3.22E-2 | 0.68 | 0.23 | 2.82E-3 | 0.015 | 0.013 | 0.24 | 0.031 | 0.024 | 0.21 | 0.009 | 0.016 | 0.56 | 0.46 | 0.21 | 3.27E-2 |
| **SA** | -0.013 | 0.014 | 0.33 | 1.67 | 0.08 | <2E-16 | 0.011 | 0.005 | 2.12E-2 | -0.038 | 0.009 | 1.45E-5 | -0.014 | 0.006 | 1.52E-2 | -0.18 | 0.08 | 2.55E-2 |

A: African, AA: African-American, EA: East Asian: API: Asian Pacific Islander, SA: South Asian, LA-1: Latin-American 1, LA-2: Latin-American-2, HDL: High-density lipoprotein cholesterol

Linear regression was used to test association of ancestry with glucose, HbA1c, triglyceride, HDL, apolipoprotein B and total fatty acids including age, centered quadratic age, WHR, menopause status, fasting hours (>3 hours vs. ≤3 hours), blood sampling hour, smoking status, alcohol intake frequency, Townsend depravation index, and summed MET minutes per week for all activities as covariates in the model.

* Individuals on lipid lowering medications were excluded from the analysis.

**Table S15:** Interaction between ancestry and WHR affecting HbA1c in females

| **Ancestry** | **Beta** | **SE** | **P** |
| --- | --- | --- | --- |
| A | 3.32 | 1.66 | 4.52E-02 |
| AA | 4.72 | 1.38 | 6.33E-04 |
| API | 5.07 | 3.07 | 0.10 |
| EA | 2.07 | 2.11 | 0.33 |
| LA-1 | 0.94 | 2.09 | 0.65 |
| LA-2 | 4.09 | 3.43 | 0.23 |
| SA | 3.06 | 1.13 | 6.86E-03 |

**Supplementary Figures**

**Figure S1:** Distribution of fasting time

| **A.** All values |
| --- |
| 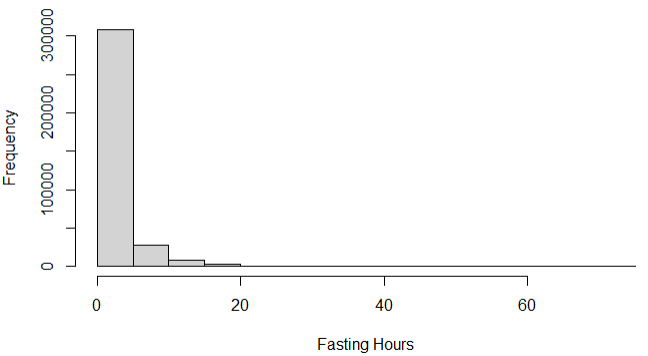 |
| **B.** Values <8 hours |
| 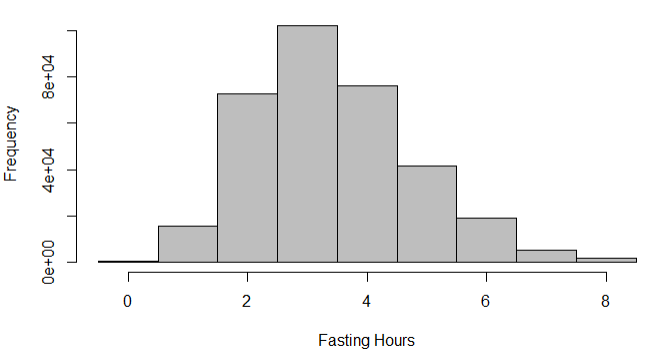 |

**Figure S2:** Distribution of anthropometric and body composition measures in males and females

| **WHR** | **Leg Fat Percentage** |
| --- | --- |
| **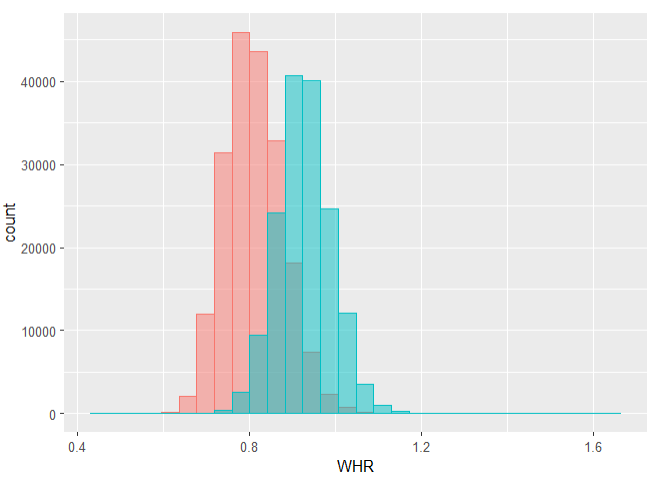** | **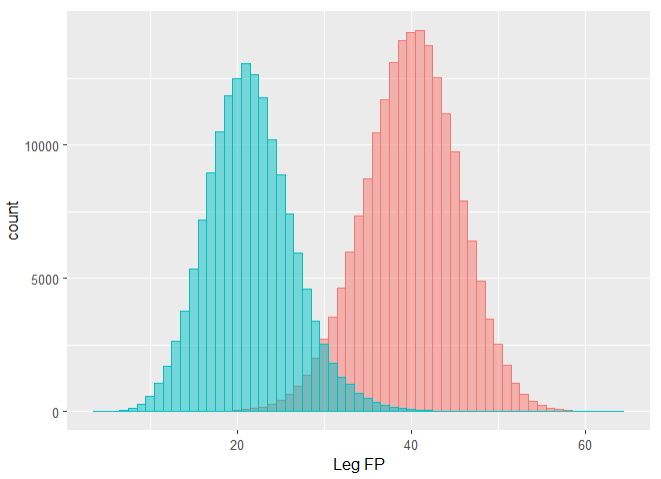** |
| **Whole Body Fat Percentage** | **Leptin** |
| **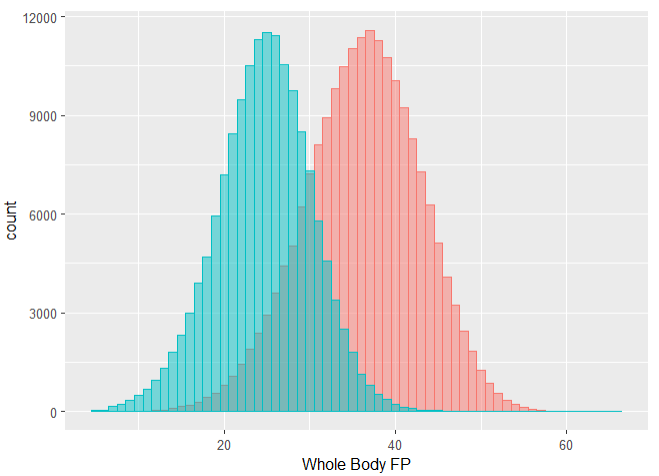** | **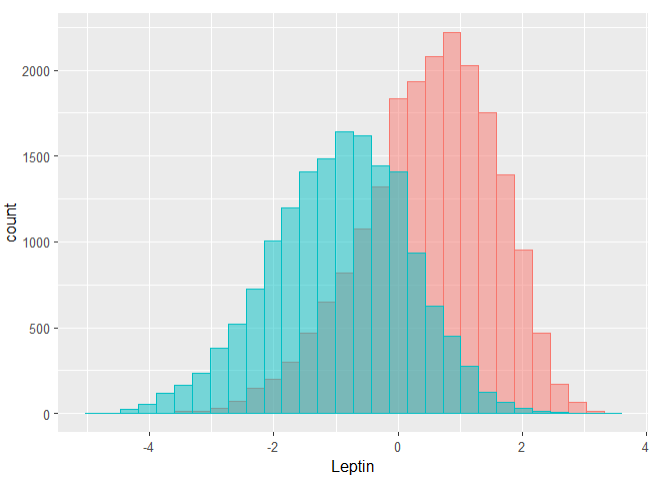** |
| **Trunk Fat Percentage** |  |
| **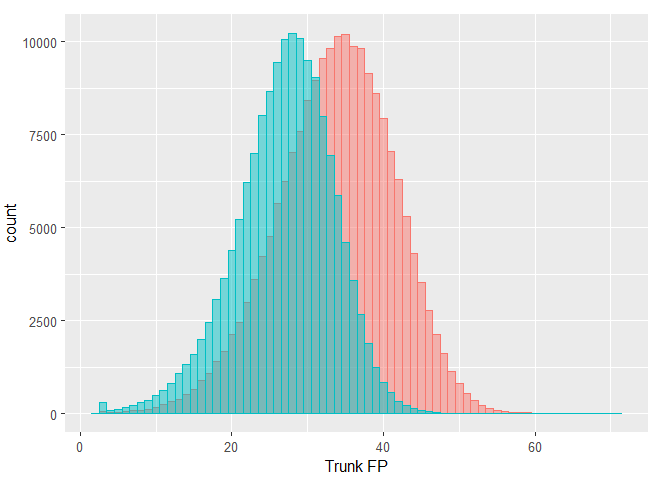** | **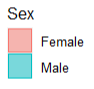** |

**Figure S3:** Distribution of glycemic and lipid traits in males and females

| **Glucose** | **HDL** |
| --- | --- |
| **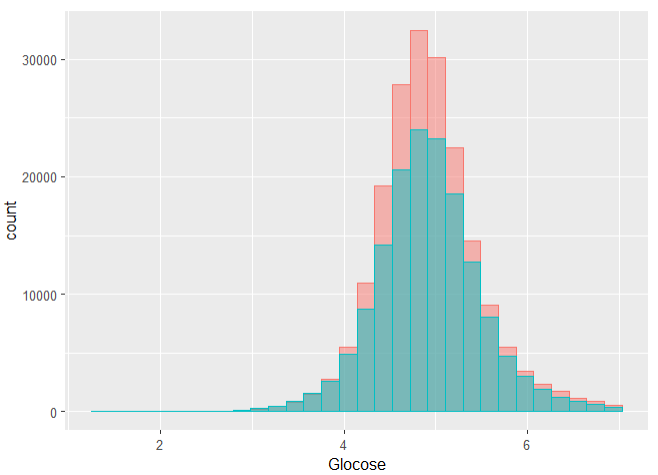** | **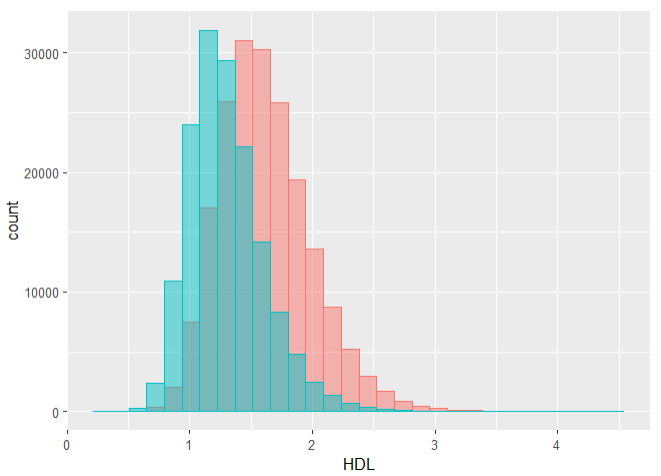** |
| **HbA1c** | **Apolipoprotein B** |
| **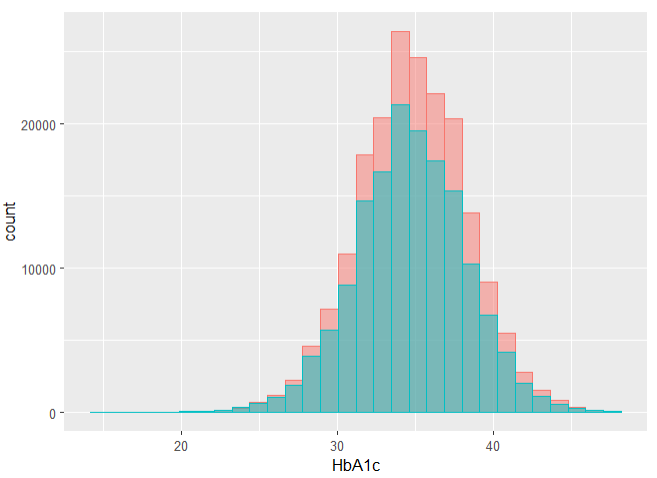** | **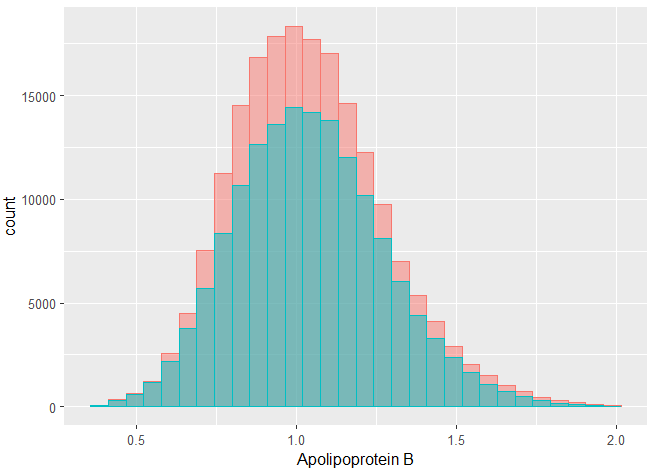** |
| **Triglyceride** | **Total Fatty Acids** |
| **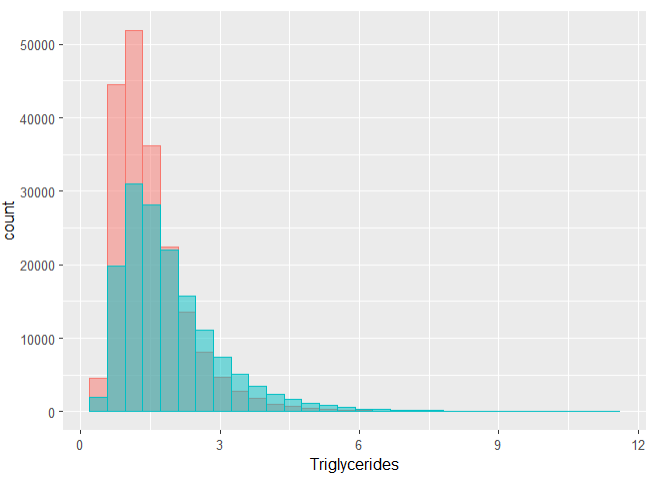** | **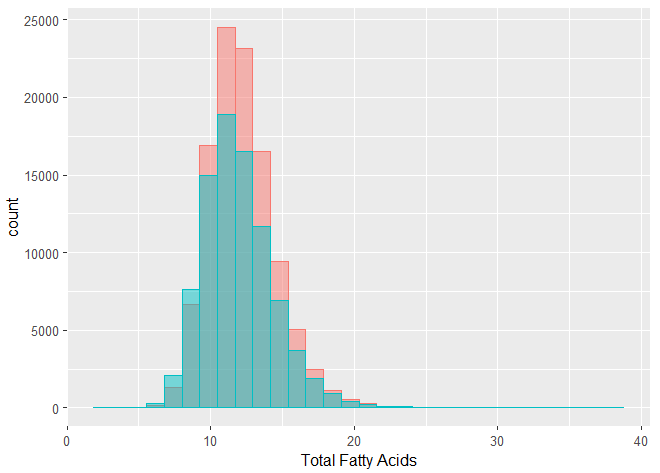** |
|  | **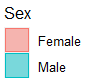** |

**Figure S4:** Genetic distance (GD) scores

| **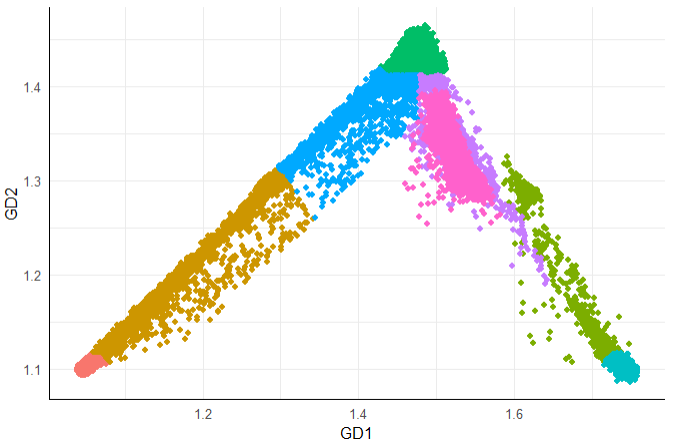** | **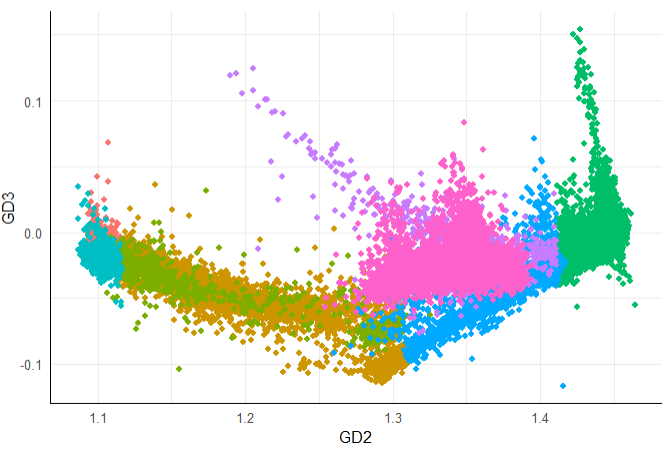** | **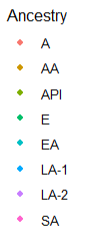** |
| --- | --- | --- |
| **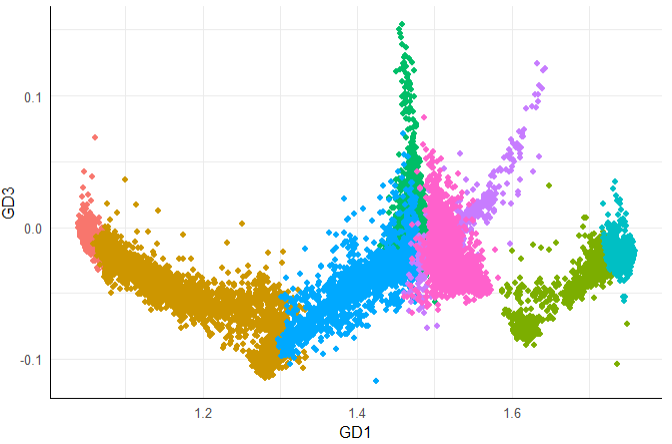** | **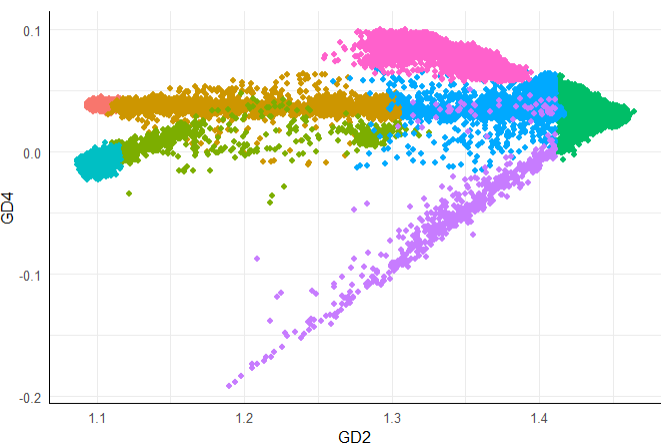** |  |
| **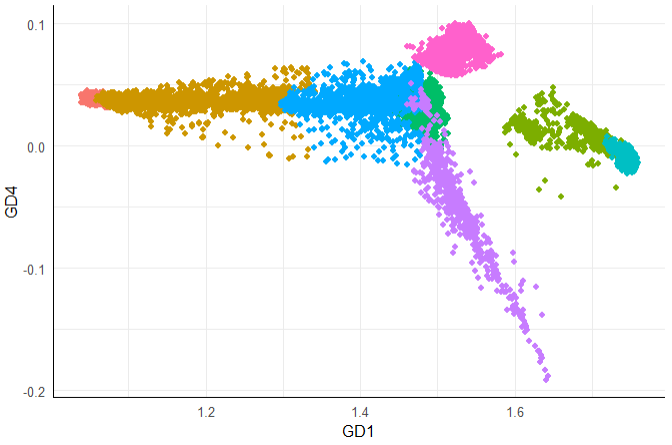** | **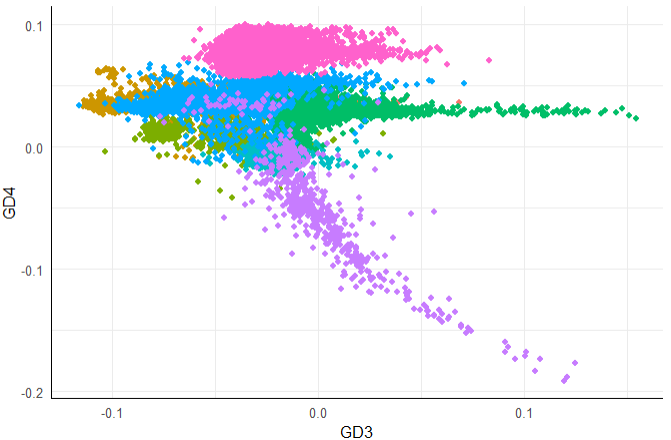** |  |

**Figure S5:** GD1 vs. GD2 in different ancestries

| 1. European | 1. African | 1. African American | 1. Asian Pacific Islanders |
| --- | --- | --- | --- |
| 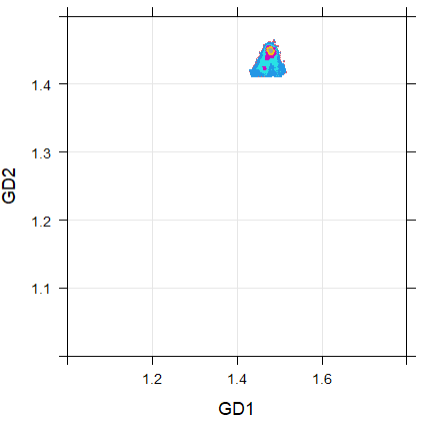 | 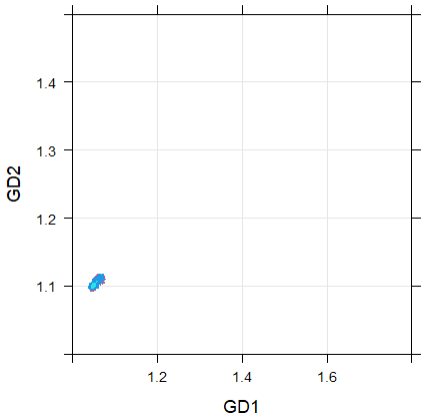 | 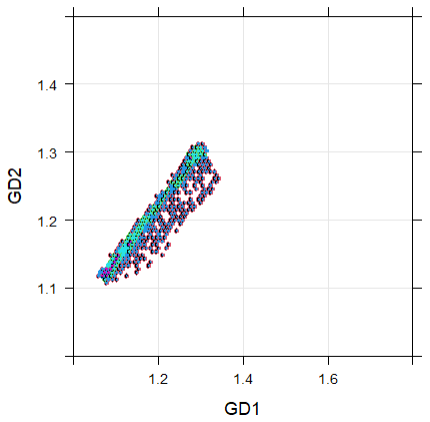 | 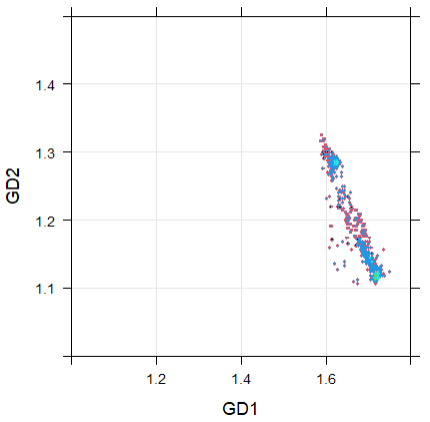 |
| 1. East Asians | 1. Latin American 1 | 1. Latin American 2 | 1. South Asian |
| 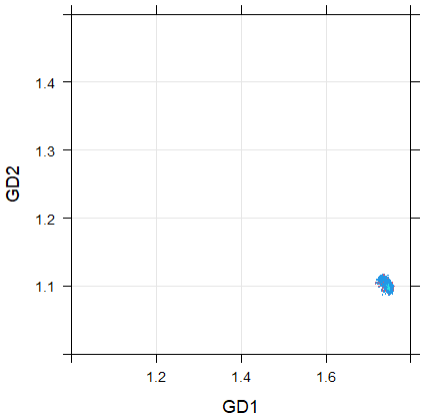 | 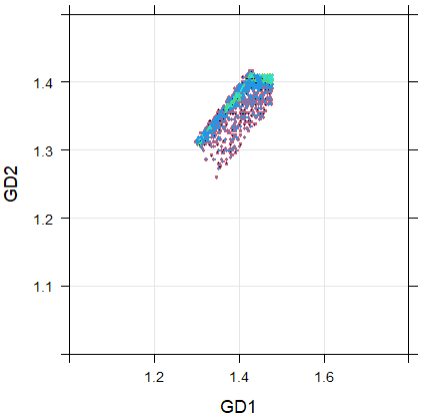 | 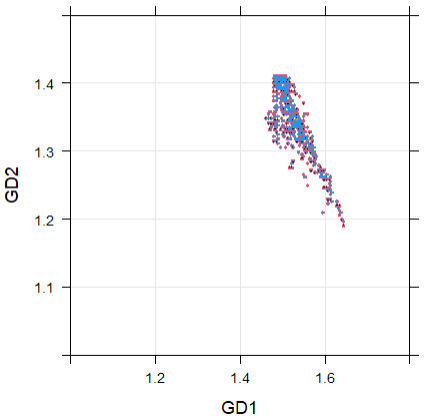 | 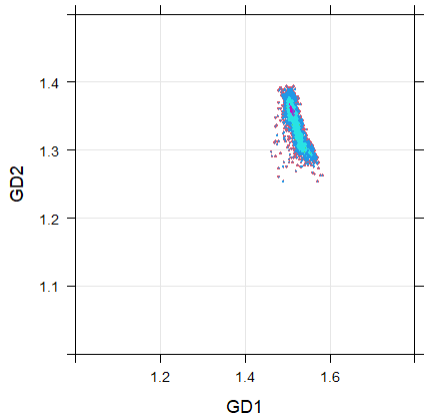 |
|  |  |  | 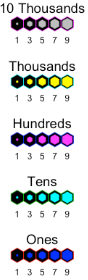 |

**Figure S6:** Fat percentages in males and females measured by impedance vs. DXA

| **Males** | **Females** |  |
| --- | --- | --- |
| **Whole body fat percentage** | |  |
| **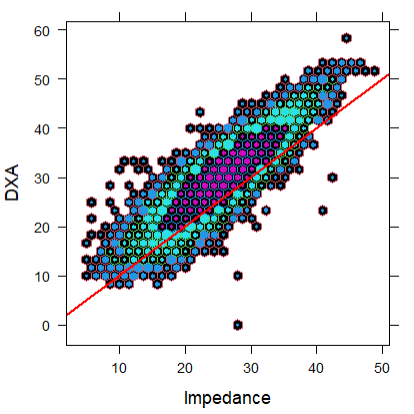** | **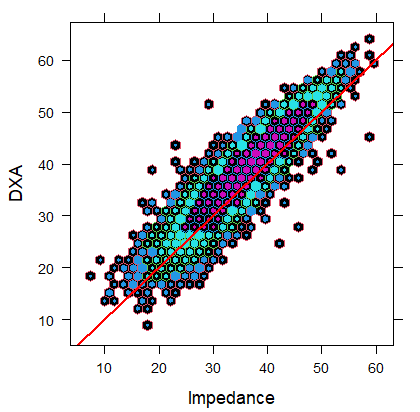** | **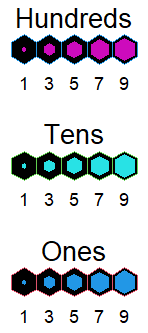** |
| **Trunk fat percentage** | |  |
| **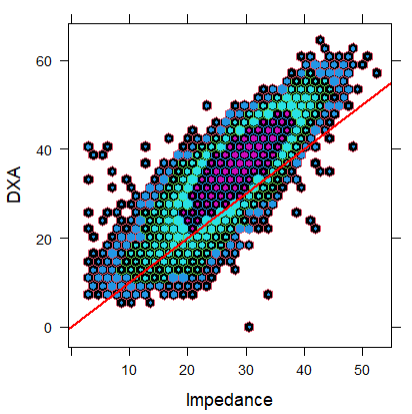** | **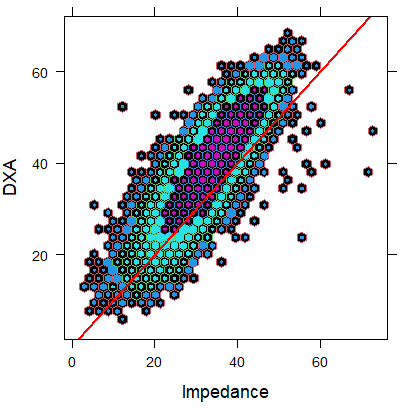** |  |
| **Leg fat percentage** | |  |
| **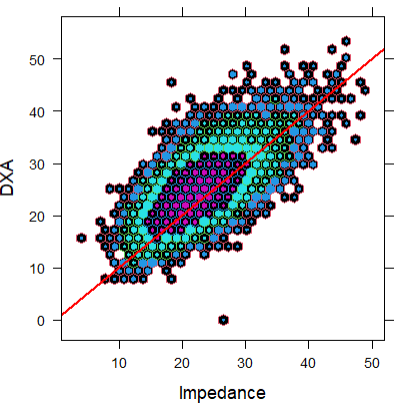** | **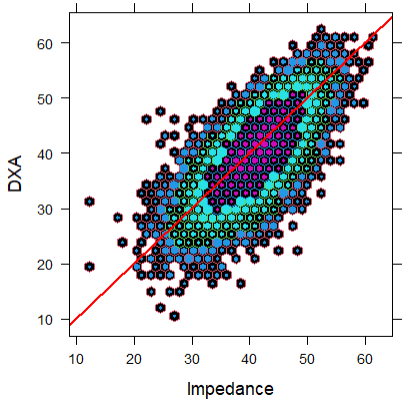** |  |

Hexbin bivariate density plots were generated to visualize overlapping points using the R packages lattice and hexbin. The red diagonal line is showing Impedance = DXA.

STROBE Statement—checklist of items that should be included in reports of observational studies

|  | Item No | Recommendation | Page N |
| --- | --- | --- | --- |
| **Title and abstract** | 1 | (*a*) Indicate the study’s design with a commonly used term in the title or the abstract | Title |
|  |  | (*b*) Provide in the abstract an informative and balanced summary of what was done and what was found | Abstract |
| Introduction | | |  |
| Background/rationale | 2 | Explain the scientific background and rationale for the investigation being reported | Section 1. |
| Objectives | 3 | State specific objectives, including any prespecified hypotheses | Section 1. |
| Methods | | |  |
| Study design | 4 | Present key elements of study design early in the paper | Section 2. |
| Setting | 5 | Describe the setting, locations, and relevant dates, including periods of recruitment, exposure, follow-up, and data collection | Section 2. |
| Participants | 6 | (*a*) *Cohort study*—Give the eligibility criteria, and the sources and methods of selection of participants. Describe methods of follow-up  *Case-control study*—Give the eligibility criteria, and the sources and methods of case ascertainment and control selection. Give the rationale for the choice of cases and controls  *Cross-sectional study*—Give the eligibility criteria, and the sources and methods of selection of participants | Section 2. |
|  |  | (*b*) *Cohort study*—For matched studies, give matching criteria and number of exposed and unexposed  *Case-control study*—For matched studies, give matching criteria and the number of controls per case | - |
| Variables | 7 | Clearly define all outcomes, exposures, predictors, potential confounders, and effect modifiers. Give diagnostic criteria, if applicable | Section 2. |
| Data sources/ measurement | 8* | For each variable of interest, give sources of data and details of methods of assessment (measurement). Describe comparability of assessment methods if there is more than one group | Section 2. |
| Bias | 9 | Describe any efforts to address potential sources of bias | Section 2. |
| Study size | 10 | Explain how the study size was arrived at | Section 2. |
| Quantitative variables | 11 | Explain how quantitative variables were handled in the analyses. If applicable, describe which groupings were chosen and why | Section 2. |
| Statistical methods | 12 | (*a*) Describe all statistical methods, including those used to control for confounding | Section 2.6. |
|  |  | (*b*) Describe any methods used to examine subgroups and interactions | Section 2.6. |
|  |  | (*c*) Explain how missing data were addressed | Section 2.6 |
|  |  | (*d*) *Cohort study*—If applicable, explain how loss to follow-up was addressed  *Case-control study*—If applicable, explain how matching of cases and controls was addressed  *Cross-sectional study*—If applicable, describe analytical methods taking account of sampling strategy | Section 2.6. |
|  |  | (*e*) Describe any sensitivity analyses | - |

Continued on next page

| Results | | |  |
| --- | --- | --- | --- |
| Participants | 13* | (a) Report numbers of individuals at each stage of study—eg numbers potentially eligible, examined for eligibility, confirmed eligible, included in the study, completing follow-up, and analysed | Section 3.  Tables 1, 2, S1 |
|  |  | (b) Give reasons for non-participation at each stage | Tables 1, 2, S1 |
|  |  | (c) Consider use of a flow diagram | - |
| Descriptive data | 14* | (a) Give characteristics of study participants (eg demographic, clinical, social) and information on exposures and potential confounders | Section 3. Tables 1, 2, S1 |
|  |  | (b) Indicate number of participants with missing data for each variable of interest | Table S1 |
|  |  | (c) *Cohort study*—Summarise follow-up time (eg, average and total amount) | - |
| Outcome data | 15* | *Cohort study*—Report numbers of outcome events or summary measures over time | - |
|  |  | *Case-control study—*Report numbers in each exposure category, or summary measures of exposure | - |
|  |  | *Cross-sectional study—*Report numbers of outcome events or summary measures | Table 1 & 2 |
| Main results | 16 | (*a*) Give unadjusted estimates and, if applicable, confounder-adjusted estimates and their precision (eg, 95% confidence interval). Make clear which confounders were adjusted for and why they were included | Section 3.  Table 3,4, S6-S9  Figure 1-2 |
|  |  | (*b*) Report category boundaries when continuous variables were categorized | - |
|  |  | (*c*) If relevant, consider translating estimates of relative risk into absolute risk for a meaningful time period | - |
| Other analyses | 17 | Report other analyses done—eg analyses of subgroups and interactions, and sensitivity analyses | - |
| Discussion | | |  |
| Key results | 18 | Summarise key results with reference to study objectives | Section 4. |
| Limitations | 19 | Discuss limitations of the study, taking into account sources of potential bias or imprecision. Discuss both direction and magnitude of any potential bias | Section 4.4. |
| Interpretation | 20 | Give a cautious overall interpretation of results considering objectives, limitations, multiplicity of analyses, results from similar studies, and other relevant evidence | Section 4. |
| Generalisability | 21 | Discuss the generalisability (external validity) of the study results | Section 4.4. and 4.5 |
| Other information | | |  |
| Funding | 22 | Give the source of funding and the role of the funders for the present study and, if applicable, for the original study on which the present article is based | Funding Section |

*Give information separately for cases and controls in case-control studies and, if applicable, for exposed and unexposed groups in cohort and cross-sectional studies.

**Note:** An Explanation and Elaboration article discusses each checklist item and gives methodological background and published examples of transparent reporting. The STROBE checklist is best used in conjunction with this article (freely available on the Web sites of PLoS Medicine at http://www.plosmedicine.org/, Annals of Internal Medicine at http://www.annals.org/, and Epidemiology at http://www.epidem.com/). Information on the STROBE Initiative is available at www.strobe-statement.org.
